# Overcoming the language barrier: a novel curriculum for training medical students as volunteer medical interpreters

**DOI:** 10.1186/s12909-021-03081-0

**Published:** 2022-01-10

**Authors:** Erik S Carlson, Tatiana M Barriga, Dale Lobo, Guadalupe Garcia, Dayana Sanchez, Matthew Fitz

**Affiliations:** 1 Stritch School of Medicine, Loyola University Chicago, Maywood, 60153 USA; 2 Department of Interpreter Services, Loyola University Medical Center, Gottlieb Memorial Hospital, MacNeal Hospital, Maywood, 60153 USA; 3 Department of Interpreter Services, Loyola University Medical Center, Maywood, 60153 USA; 4 Department of Medicine, Loyola University Medical Center, Stritch School of Medicine, Loyola University Chicago, Maywood, 60153 USA

**Keywords:** Undergraduate Medical Education, Medical Student, Medical Spanish, Interpreter, Volunteer, Charitable clinic, Resident continuity clinic

## Abstract

**Background:**

Over 41 million people in the United States speak Spanish as their primary language, of which 16 million have limited English proficiency (LEP). It is well-established that language barriers contribute to health disparities and that the use of ad-hoc interpretation by untrained family members results in substandard care. We developed a novel interpreter training program for medical students to serve as in-person interpreters at a charitable, resident continuity clinic so as to overcome the language barrier in the delivery of healthcare to LEP patients.

**Methods:**

The Medical Student Interpreter Training Program (MSITP) consists of three steps. First, fluent Spanish-speaking students shadowed a licensed interpreter. Second, students took a standardized phone exam to demonstrate language proficiency. Finally, students completed a three-hour training on the methodology and ethics of interpreting conducted by the Department of Interpreter Services.

**Results:**

Pre- and post-tests were administered to assess students’ familiarity with the Interpreter Code of Ethics and interpreter skills. Familiarity with the Interpreter Code of Ethics increased significantly with all students reporting feeling comfortable (47%) or very comfortable (53%) after training. The pre- and post-tests included free response questions, which were administered to assess competence in the methodology and ethics of interpreting. The cohort’s aggregate score increased by 35% after the training (Wilcoxon signed rank z-score = 2.53; *p* = .01).

**Conclusions:**

Implementing the MSITP resulted in an increased number of trained, Spanish-speaking interpreters available to provide their services to LEP patients at an affiliated charitable clinic and throughout the university hospital. Unlike other program models which are time and resource-intensive, this program is replicable and easily managed by volunteers. The MSITP is an effective model for training students as medical interpreters to ensure the delivery of quality healthcare for LEP patients.

**Supplementary Information:**

The online version contains supplementary material available at 10.1186/s12909-021-03081-0.

## Background

As of 2018, over 41 million people in the United States speak Spanish as their primary language, and approximately 16 million of those persons state that they speak English less than “very well.” [1] This large number of patients with limited English proficiency (LEP) comes with a corresponding need for medical interpreters. Because language barriers contribute to health disparities, trained interpreters are essential for bridging the linguistic and cultural divide between patients with LEP and their healthcare providers [2–11]. Despite policies requiring otherwise, it is relatively common practice for physicians that are treating LEP patients to turn to bilingual family members or untrained, ostensibly bilingual staff for interpretation when a licensed interpreter is not immediately available [12, 13]. Such occurrences happen regardless of whether or not phone interpreter infrastructure is in place because providers and patients tend to prefer in-person medical interpreters over phone interpretation [14–16]. Additionally, previous research demonstrating that providers' self-reported Spanish proficiency does not reliably predict tested ability further reinforces the need for a structured language credentialing to improve care for LEP patients [17].

Although Title VI of the Civil Rights Act of 1964 requires that all recipients of federal financial assistance provide language services to all LEP persons to ensure quality healthcare, there remains a limited supply of bilingual providers or interpreters who can help overcome cultural and language barriers in the delivery of healthcare [18, 19]. In response to the increasing need for bilingual providers, a few medical schools have sought to develop interpreter training programs for medical students [20–24]. Ideally, these interpreter trained students can assist in language appropriate care through volunteering in their university hospital and at affiliated free or charitable clinics [11, 25].

In reviewing formal training programs for interpreters, we identified that current interpreter training programs range from two to two hundred hours in length and vary in their rigor and comprehensiveness [26]. Given the time constraints and competing demands of medical students' schedules, we sought to identify an interpreter training program that would be practical and realistic to implement without sacrificing interpreter standards of care. Upon review of potential interpreter training programs for medical students, we found pre-existing program models to be too time and resource-intensive for medical students and faculty [20–24]. Additionally, we discovered other pre-existing interpreter program models not specific enough to the needs of the Spanish-speaking population which our university’s affiliated charitable, resident continuity clinic serves [20, 21, 23]. Thus, we instead sought to leverage the pre-existing resources of the university hospital's department of interpreter services to establish a time-efficient and replicable interpreter training program for medical students.

After multiple pilot programs and a review of similar programs implemented at other institutions, we decided upon a three-step Medical Student Interpreter Training Program (MSITP) with the intent of being feasible and replicable at any healthcare facility with a department of interpreter service and medical student volunteers. This program provides a framework for future medical student interpreter programs and helps overcome the barriers to patient safety created by the ad-hoc use of untrained medical students or family members as interpreters.

## Methods

In developing the MSITP, we recruited only first and second-year medical students as well as graduate students who self-identified as fluent in Spanish. Our program could not offer the resources necessary to teach students a language in addition to training them as interpreters. Thus, we took advantage of our medical school’s population of Spanish-speaking students with the goal of training students so that they could interpret for patients.

The MSITP consists of a three-step process: (1) shadowing a licensed hospital interpreter for one hour (2) taking the ALTA Language Services Qualified Bilingual Staff (QBS) Assessment via telephone, a standardized phone exam which consists of 5 sections: conversation/social, customer service, nursing diagnosis and instructions, medical terminology, and sight translation, [27] and (3) attending a one-time, three-hour interpreter instruction session conducted by the department of interpreter services, which includes multiple practice scenarios and an informal evaluation of the trainees' interpretation skills.

To advance to the interpreter training session, students must first achieve a level two status on the standardized QBS phone exam, which means scoring a 70% or above in each of the four sections. After attaining a level two status, the students take part in a 3-h interpreter training held by interpreter services. If students achieve a level one on the QBS phone exam, meaning that they had an objective score of 70% and higher and a subjective rating of 3 or higher in customer services, they have the option to repeat the exam at a later date. If a student does not achieve a level one, meaning that they had an objective score of lower than 70%, then they cannot progress further in the interpreter program.

Before the 3-h interpreter training session, students pre-read the National Council on Interpreting in Health Care’s National Code of Ethics for Interpreters in Healthcare and National Standards of Practice for Interpreters in Healthcare [28, 29]. Upon arrival to the training session, students completed the Interpreter Training Pre-test (Additional file A). During the first 90 min of the training session, the instructor reviewed the Department of Interpreter Services' Qualified Bilingual Staff Program training slides (Additional file B), which covered each of the program’s educational objectives.

Following the completion of the presentation, the instructor divided the students into small groups, at which point they practiced interpreter scenarios sourced from a textbook provided by the Department of Interpreter Services [30]. Students alternated between the role of the patient, the provider, and the interpreter as they practiced the scenarios. While practicing the role of the interpreter, the professional interpreter and the student observers filled out the QBS Evaluation Form (Additional file C) independently to provide each student with constructive feedback on their interpretation. Once each student had played the role of the patient, provider, and interpreter to the satisfaction of the professional interpreter and student observers, the professional interpreters and student observers then provided constructive feedback to the student interpreter by means of the QBS Evaluation Form. After the students had reviewed their feedback, they then completed the Interpreter Training Post-Test (Additional file D).

Following successful completion of the three-hour training session, students were provided with a QBS Level 2 ID Badge (Additional file E) to hang behind their photo identification card. This badge qualifies the student to provide services in the Spanish language in a variety of healthcare settings across the university hospital system and specifically at the university’s affiliated charitable, resident continuity clinic. Furthermore, this badge allows faculty and staff to recognize these students as trained medical Spanish interpreters. However, students are still instructed to know their limitations and call a licensed interpreter in more challenging settings, such as informed consents, palliative care, and encounters involving the police (Additional file F).

We developed and implemented the Interpreter Training Exercise Pre-Test (Additional file A) and Post-Test (Additional file D) as a means of assessing the students’ understanding of the Interpreter Code of Ethics and appropriate techniques of interpreting for LEP patients, both of which were critical to the realization of the program’s goals of providing adequate and safe interpretation for patients. These were novel scoring instruments because an extensive review of the literature did not reveal any standardized questionnaires that would measure these outcomes. Short, essay-style questions were implemented in the assessment as they made it possible to assess a student’s complex thinking and their ability to integrate their assigned reading of the National Code of Ethics for Interpreters in Health Care, [28] the National Standards of Practice for Interpreters in Healthcare, [29] the presentation given at the beginning of the training session (Additional file B), and the role-playing exercises.

Independent staff from the hospital’s Department of Interpreter Services graded the interpreter training pre- and post-assessments as they are subject matter experts. Our senior faculty member deidentified the assessments prior to their being graded, and the interpreters grading the tests were blinded as to whether they were grading a pre- or post- assessment. We designed the questions and the methods for grading the assessments using guidelines outlined by the *Best Practices for Designing and Grading Exams* rubric from the University of Michigan’s Center for Research on Learning and Teaching [31]. We generated the Interpreter Pre & Post Test Free Response Grading Rubric (Additional file G) for the subject matter experts to follow when grading the tests. The rubric included the questions followed by the criteria needed to be met for an answer to receive full credit. Each question was assigned a range of points that could be allocated to a given response because some of the free responses required multiple criteria for a perfect score. The independent interpreter staff graded one question at a time in accordance with the rubric (e.g. to grade all of the question #1’s before moving on to grade the question #2’s). They used their best professional judgement if the answers were not verbatim with the answer key. All responses were analyzed using Microsoft Excel 2019. Descriptive statistics were used to analyze the pre- and post-assessments. We compared improvement in the distribution of scores from pre-test to post-test using the Wilcoxon signed-rank test.

## Results

### Student Population and Prior Experiences

Since the implementation of the program in the fall of 2018, twenty-four students have taken the QBS phone exam, with a pass rate of 71% (Table 1). Of the students that did not pass, five of the seven achieved level 1 status on the QBS phone exam, and they intend to retake the exam after reviewing their medical Spanish vocabulary (Table 1). Of the 24 students recruited, 71% passed the Level 2 QBS phone Exam, while 21% did not pass and achieved Level 1 with the intent to retake the exam at a later date (Table 1).

**Table 1:** Participant Demographics

	**2018 – 2019 Cohort (*n* = 11)**	**2019 – 2020 Cohort (*n* = 13)**	**Total** **(*n* = 24)**
**Sex**			
Male	4	6	10 (42%)
Female	7	7	14 (58%)
**Training level**			
Year 1 MD students	10	8	18 (75%)
Year 2 MD students	1	4	5 (21%)
Graduate students	0	1	1 (4%)
**QBS Phone Exam**			
Passed Level 2	9 (82%)	8 (62%)	17 (71%)
Did not pass Level 2 but achieved Level 1	2 (18%)	3 (23%)	5 (21%)
Failed QBS phone exam	0 (0%)	2 (15%)	2 (8%)

The 17 student participants were a mix of native fluent speaker (65%) and non-native fluent speakers (35%) (Table 2). All students had previously spent time as an informal interpreter with the majority of students having spent over 15 h interpreting (76%) and the remainder of students having spent six to ten hours interpreting (24%) (Table 2).

**Table 2:** Survey Responses of Students who Attended the Interpreter Training Session

	**2018 – 2019 Cohort (*n* = 9)**	**2019 – 2020 Cohort** **(*n* = 8)**	**Total** **(*n* = 17)**
**Language Proficiency**			
Native fluent speakers	8 (89%)	3 (38%)	11 (65%)
Non-native fluent speakers	1 (11%)	5 (63%)	6 (35%)
**Level of Comfort with Spanish-Speaking Skills**			
Somewhat comfortable	1 (11%)	2 (25%)	3 (18%)
Very comfortable	8 (89%)	6 (75%)	14 (82%)
**Previous Time Spent as an Informal Interpreter**			
6–10 h	3 (33%)	1 (13%)	4 (24%)
11–15 h	0 (0%)	0 (0%)	0 (0%)
> 15 h	6 (67%)	7 (88%)	13 (76%)

### Effect of Training on Student’s Comfort and Familiarity with Interpreter-skills

**Fig. 1:**
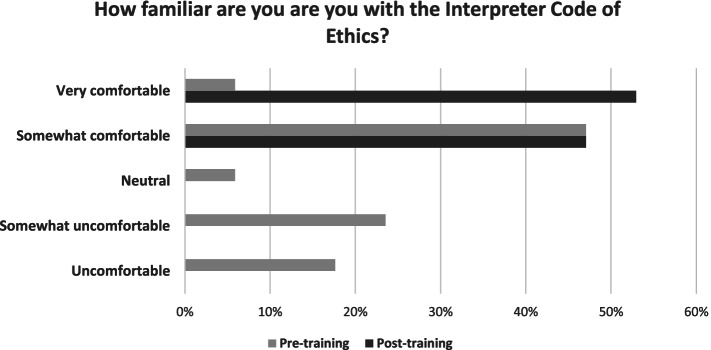
Percentage of students who self-rated familiarity with the Interpreter Code of Ethics on pre-test and post-test (*n* = 17)

After training, all students felt somewhat comfortable (47%) or very comfortable (53%) with the Interpreter Code of Ethics (Fig. 1). Students experienced more familiarity with the concept of intervening with transparency, with 41% of students feeling somewhat comfortable and 59% of students feeling very comfortable. Furthermore, the training led to an increase in the percentage of students who rated their comfort with their interpreter skills as very comfortable from pre-test (41%) to post-test (53%).

**Fig. 2:**
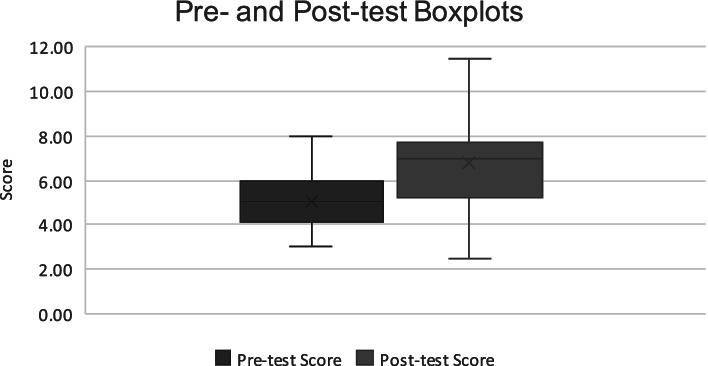
Boxplot of score distribution from Interpret Training Exercise Pre- and Post-Examinations (*n* = 17)

On evaluation of the students’ free response portion of the pre- and post-tests, we observed a 35% improvement in the cohort’s average scores before and after the training. The cohort’s average score after the training was 6.75, compared to 5.01 before the training. At pre-test, the median number of points earned was 5 (IQR: 4.25 – 6.00). At post-test, the median number of points earned increased to 7.00 (IQR: 5.25 – 7.50) which was a statistically significant increase (Wilcoxon signed rank z-score = 2.53; exact *p* = 0.01) (Fig. 2).

Seventeen students have now successfully completed the MSITP. These students received their QBS Level 2 ID Badges and have volunteered as interpreters at the university’s affiliated charitable, resident continuity clinic, which serves approximately 1,000 patients, 80% of which list Spanish as their primary language.

## Discussion

Previous iterations of the MSITP program at our institution had opened with the interpreter training followed by the QBS phone exam. In one of these instances, twenty-eight students attended the first interpreter training. While this figure was initially quite encouraging, only a small portion of those students went on to take the QBS phone exam and complete the MSITP. By restructuring the MSITP such that shadowing a professional interpreter and taking the QBS phone exam are the first steps, these steps acted as a screener, ensuring that only students who are (1) committed to completing the MSITP and (2) fluent in Spanish can progress in the interpreter training process.

From our review of previous program initiatives, we consistently found that the most significant barrier to the successful completion of the program was the time commitment required of students and faculty. In anticipation of this challenge, we streamlined the training process such that a medical student volunteer can coordinate with interpreter services to schedule a potential program candidate’s shadowing, phone exam, and training session. Thus, we created a program that is replicable at any healthcare teaching facility with a department of interpreter services and medical student volunteers. At the end of our program, students exhibited increased confidence and enhanced proficiency in the skills required to interpret safely and effectively. They also demonstrated a more comprehensive understanding of the role of the interpreter in the clinical encounter and how to intervene with transparency when necessary.

The principal limitation of this program is that we developed it to train student interpreters to work specifically with a Spanish-speaking patient population. It is conceivable that our methods and application may not be transferable to other LEP patients. Nevertheless, given the growing United States’ population of patients with limited English proficiency whose primary language is Spanish, [32] we believe this program should have good generalizability in many cities throughout the country. Additionally, the MISITP program structure can be adapted to meet the needs of a variety of patient populations. The ALTA Language Services QBS Assessment is available in 30 languages, and healthcare interpreting scenarios are available for purchase in a multitude of languages [27]. Depending upon the resources of the affiliated hospital’s department of interpreter services, the MSITP program can be adapted to any number of languages to meet the needs of a specific patient population.

Additionally, we did not formally survey our patients nor our interpreter services team. In the future, we intend to study whether our Spanish-speaking patients prefer having a medical student interpreter rather than the standard telephone interpreter. We will also survey our interpreter services team to determine if we are utilizing their time and resources more efficiently.

In making this program mandatory for all potential medical student interpreters, we hope to improve the level of patient safety and quality of care for the Spanish speaking LEP patients at our affiliated charitable, resident continuity clinic site and throughout the university hospital. We believe that this program can be a model for other healthcare teaching institutions that seek to provide a higher quality of care for LEP patients. We also demonstrated that this program can and will contribute to the development of well-trained, linguistically, and culturally humble healthcare providers.

## Supplementary Information

**Additional file 1**. Interpreter Training Pre-Test.

**Additional file 2**. Qualified Bilingual Staff Training Slides.

**Additional file 3**. Qualified Bilingual Staff Evaluation Form.

**Additional file 4**. Interpreter Training Post-Test.

**Additional file 5**. Example Qualified Bilingual Staff ID Badges.

**Additional file 6**. Description of QBS Level 1 vs. Level 2.

**Additional file 7**. Interpreter Pre & Post Test Free Response Grading Rubric.

## Data Availability

The datasets generated and/or analyzed during the current study are not publicly available due to their ability to compromise the privacy of the study participants but are available from the corresponding author on reasonable request.

## Abbreviations

LEP: Limited English proficiency
MSITP: Medical Student Interpreter Training Program
QBS: Qualified Bilingual Staff
MD: Medicinae Doctor, Doctor of Medicine

## References

[1] U.S. Census Bureau. Place of Birth by Language Spoken at Home and Ability to Speak English in the United States: 2018: ACS 1-Year Estimates Detailed Tables. Accessed August 12, 2020. https://data.census.gov/cedsci/table?t=Language%20Spoken%20at%20Home&tid=ACSDT1Y2018.B06007&hidePreview=false

[2] Casey MM, Blewett LA, Call KT (2004). Providing health care to Latino immigrants: Community-based efforts in the rural Midwest. Am J Public Health.

[3] Karliner LS, Jacobs EA, Chen AH, Mutha S (2007). Do professional interpreters improve clinical care for patients with limited English proficiency? A systematic review of the literature. Health Serv Res.

[4] Flores G, Abreu M, Barone CP, Bachur R, Lin H (2012). Errors of medical interpretation and their potential clinical consequences: a comparison of professional versus ad hoc versus no interpreters. Ann Emerg Med.

[5] Flores G, Abreu M, Olivar MA, Kastner B (1998). Access barriers to health care for Latino children. Arch Pediatr Adolesc Med.

[6] Flores G (2005). The impact of medical interpreter services on the quality of health care: a systematic review. Med Care Res Rev.

[7] Ngo-Metzger Q, Sorkin DH, Phillips RS (2007). Providing high-quality care for limited English proficient patients: the importance of language concordance and interpreter use. J Gen Intern Med.

[8] Brooks K, Stifani B, Batlle HR, Nunez MA, Matthew Erlich M, Phil M (2016). Patient perspectives on the need for and barriers to professional medical interpretation. R I Med J.

[9] Woloshin S, Bickell NA, Schwartz LM, Gany F, Welch HG (1995). Language barriers in medicine in the United States. JAMA.

[10] Nápoles AM, Santoyo-Olsson J, Karliner LS, Gregorich SE, Pérez-Stable EJ (2015). Inaccurate language interpretation and its clinical significance in the medical encounters of Spanish-speaking Latinos. Med Care.

[11] Aitken G (2019). Medical Students as Certified Interpreters. AMA J Ethics.

[12] Lee KC, Winickoff JP, Kim MK (2006). Resident physicians' use of professional and nonprofessional interpreters: a national survey. JAMA.

[13] Ryan AT, Fisher C, Chiavaroli N (2019). Medical students as interpreters in health care situations: “... it’s a grey area”. The Australian Journal of Medicine.

[14] Nápoles AM, Santoyo-Olsson J, Karliner LS, O’Brien H, Gregorich SE, Pérez-Stable EJ (2010). Clinician ratings of interpreter mediated visits in underserved primary care settings with ad hoc, in-person professional, and video conferencing modes. J Health Care Poor Underserved.

[15] Garcia EA, Roy LC, Okada PJ, Perkins SD, Wiebe RA (2004). A comparison of the influence of hospital-trained, ad hoc, and telephone interpreters on perceived satisfaction of limited English-proficient parents presenting to a pediatric emergency department. Pediatr Emerg Care.

[16] Crossman KL, Wiener E, Roosevelt G, Bajaj L, Hampers LC (2010). Interpreters: telephonic, in-person interpretation and bilingual providers. Pediatrics.

[17] Lion KC, Thompson DA, Cowden JD (2013). Clinical Spanish use and language proficiency testing among pediatric residents. Acad Med.

[18] Bureau of Labor Statistics USDoL. Job Outlook - Interpreters and Translators. Accessed July 24, 2019. https://www.bls.gov/ooh/media-and-communication/interpreters-and-translators.htm#tab-6

[19] Title VI, Prohibition Against National Origin Discrimination Affecting Limited English Proficient Persons, 42 U.S. Code § 2000d (1964).

[20] Ono N, Kiuchi T, Ishikawa H (2013). Development and pilot testing of a novel education method for training medical interpreters. Patient Educ Couns.

[21] Pelaez AFV, Ramirez SI, Sanchez CV (2018). Implementing a medical student interpreter training program as a strategy to developing humanism. BMC Med Educ.

[22] Diaz JE, Ekasumara N, Menon NR (2016). Interpreter training for medical students: pilot implementation and assessment in a student-run clinic. BMC Med Educ.

[23] Avalos OH, Pennington K, Osterberg L (2013). Revolutionizing volunteer interpreter services: an evaluation of an innovative medical interpreter education program. J Gen Intern Med.

[24] Highland J, Enriquez B, Lowenstein SR (2019). The Impact of a Student-Taught Course in Spanish Language Interpreting on Patient Care at a Student-Run Free Clinic. Journal of Student-Run Clinics.

[25] Piontek ME. *Best Practices for Designing and Grading Exams*. University of Michigan; 2008. CRLT Occasional Papers.

[26] The National Council on Interpreting in Health Care. *National Standards for Healthcare Interpreter training Programs*. 2011. https://www.ncihc.org/

[27] ATLA Language Services. Qualified Bilingual Staff Assessment. Accessed July 24, 2019. https://www.altalang.com/language-testing/qbs/

[28] The National Council on Interpreting in Health Care. *A National Code of Ethics for Interpreters in Healthcare*. 2004. https://www.ncihc.org/

[29] The National Council on Interpreting in Health Care. *National Standards of Practice for Interpreters in Health Care*. 2005. https://www.ncihc.org/

[30] International Language Services Inc. *Healthcare Interpreting Practice Dialogues, English - Spanish, Volume 1*. 2010.

[31] Piontek ME. Best Practices for Designing and Grading Exams. CRLT Occasional Papers. Ann Arbor, MI: University of Michigan; 2008.

[32] U.S. Census Bureau. Language Spoken at Home by Ability to Speak English for the Population 5 Years and Over: 2013–2017 American Community Survey 5-Year Estimates. Accessed July 20, 2019. https://factfinder.census.gov/faces/nav/jsf/pages/index.xhtml

