# Supplementary material for: Overcoming the language barrier: a novel curriculum for training medical students as volunteer medical interpreters
Source: BMC Med Educ. 2022 Jan 10;22:27. doi: 10.1186/s12909-021-03081-0 (PMC8751325; doi:10.1186/s12909-021-03081-0)
Supplement: Supplementary file 1 — Additional file 1. Interpreter Training Pre-Test. [file 12909_2021_3081_MOESM1_ESM.pdf]

# Interpreter Training Exercise

# Pre-Test

Name: ________________________________

Date: _________________________

1. Age: ________
2. Sex:
    - Male
    - Female
3. Country of Birth: ___________________
4. Country of Citizenship: __________________
5. Native speaker?
    - Yes
    - No
6. How much time, approximately, have you informally served as an interpreter:
    - 0 hours
    - 1-5 hours
    - 6-10 hours
    - 11-15 hours
    - >15 hours

7. How comfortable do you feel in your Spanish-speaking skills?
    - Uncomfortable
    - Somewhat comfortable
    - Neutral
    - Somewhat comfortable
    - Very comfortable

8. How comfortable do you feel in your interpreter skills?
    - Uncomfortable
    - Somewhat comfortable
    - Neutral
    - Somewhat comfortable
    - Very comfortable

9. How familiar are you with the Interpreter Code of Ethics?
    - Unfamiliar
    - Somewhat unfamiliar
    - Neutral
    - Familiar
    - Very familiar

10. How familiar are you with the concept of Intervening with Transparency?
    - Unfamiliar
    - Somewhat unfamiliar
    - Neutral
    - Familiar
    - Very familiar

Pre-Test Assessment

1. During the encounter, the attending doctor holds a side conversation with one of his residents. The patient looks at you, the interpreter, expecting for you to interpret the doctors' conversation, briefly explain how you should handle this situation?

2. Describe one of the principles of the interpreter code of ethics?

3. Why should the interpreter maintain the register of the speaker when interpreting?

4. List the barriers to interpreting in healthcare.

5. List the steps to intervening with transparency.
