# Supplementary material for: Overcoming the language barrier: a novel curriculum for training medical students as volunteer medical interpreters
Source: BMC Med Educ. 2022 Jan 10;22:27. doi: 10.1186/s12909-021-03081-0 (PMC8751325; doi:10.1186/s12909-021-03081-0)
Supplement: Supplementary file 2 — Additional file 2. Qualified Bilingual Staff Training Slides. [file 12909_2021_3081_MOESM2_ESM.pptx]

## Slide 1
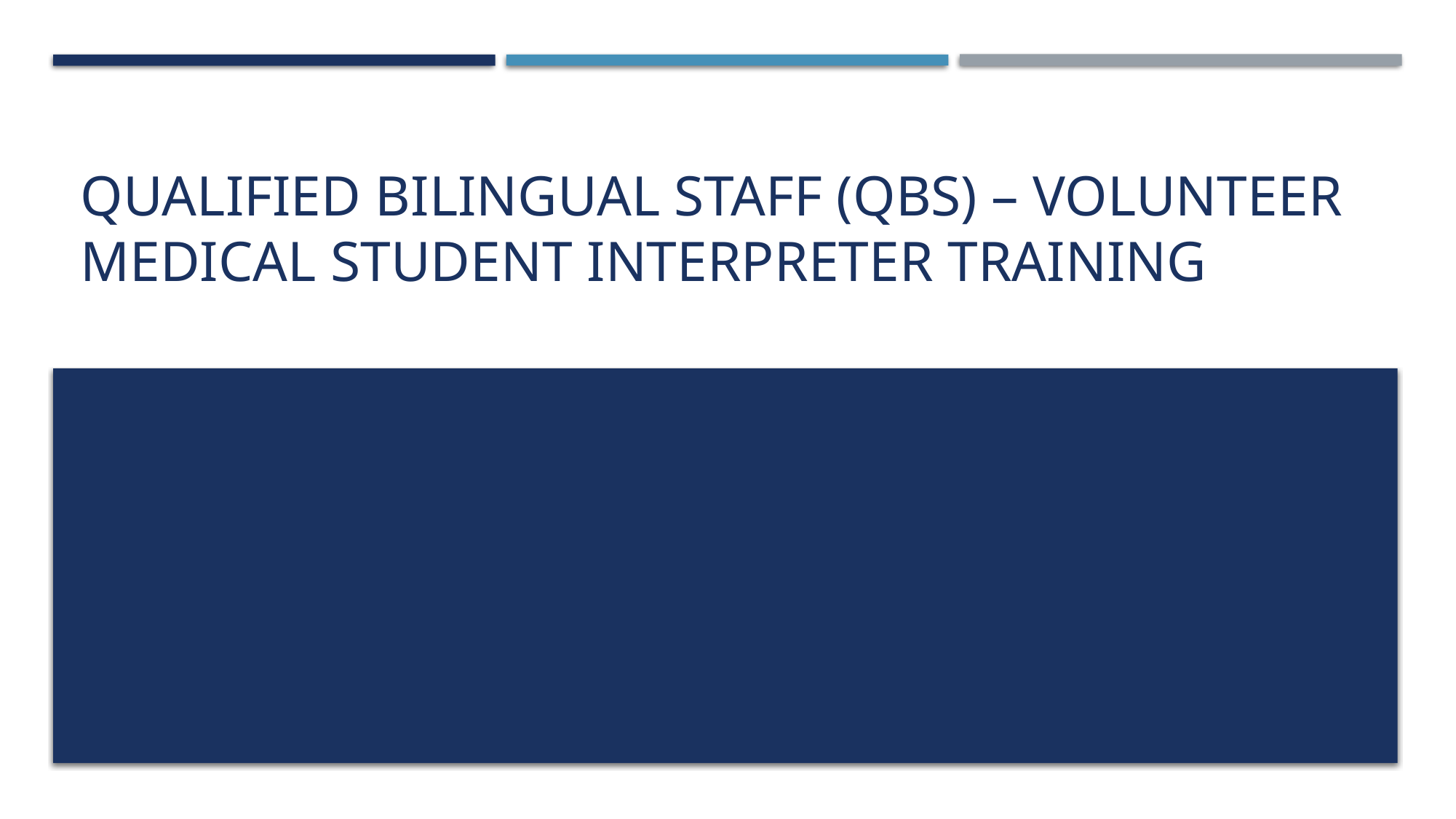

# Qualified Bilingual Staff (QBS) – Volunteer medical student Interpreter Training

## Slide 2
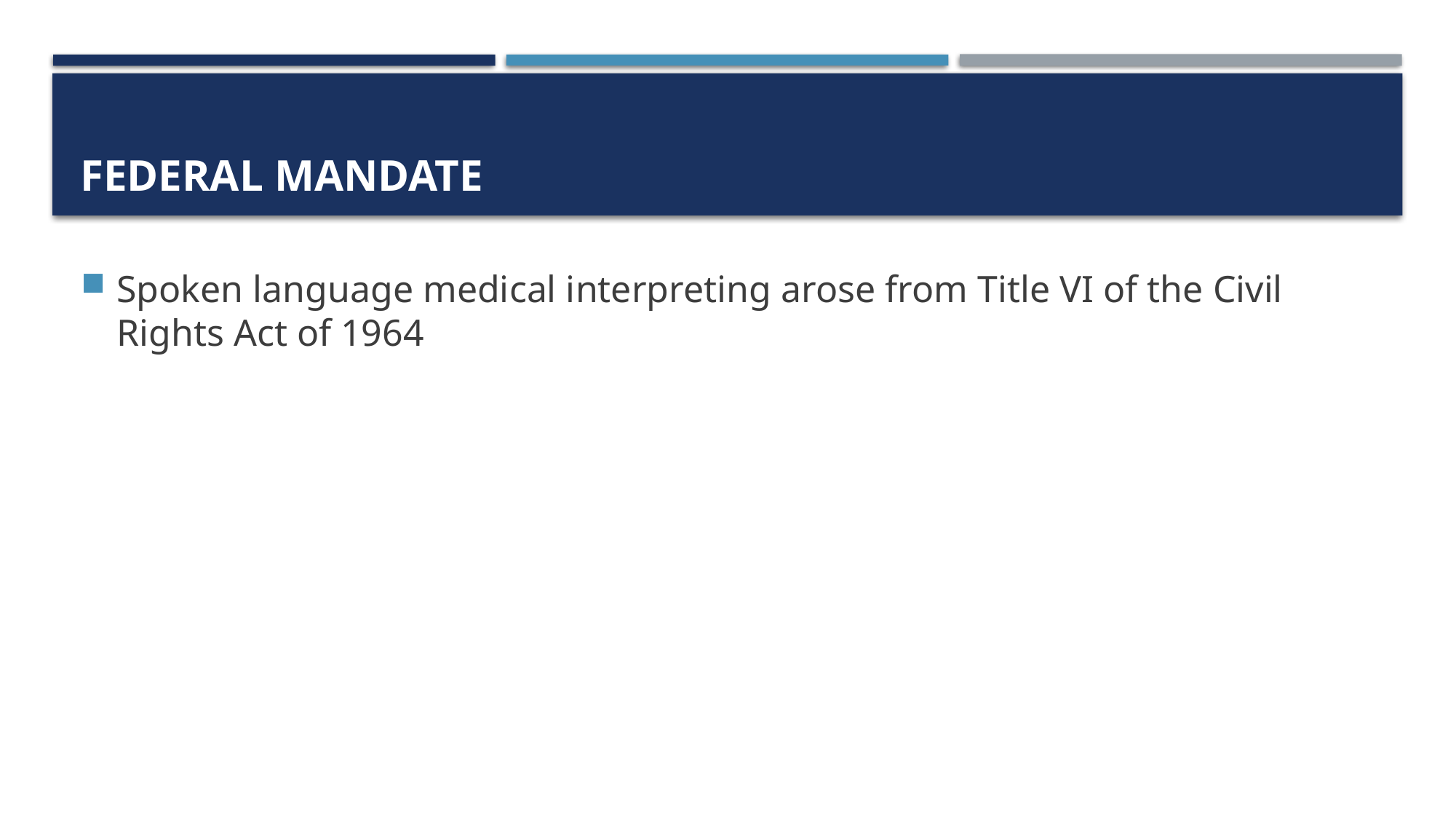

# Federal Mandate
Spoken language medical interpreting arose from Title VI of the Civil Rights Act of 1964

## Slide 3
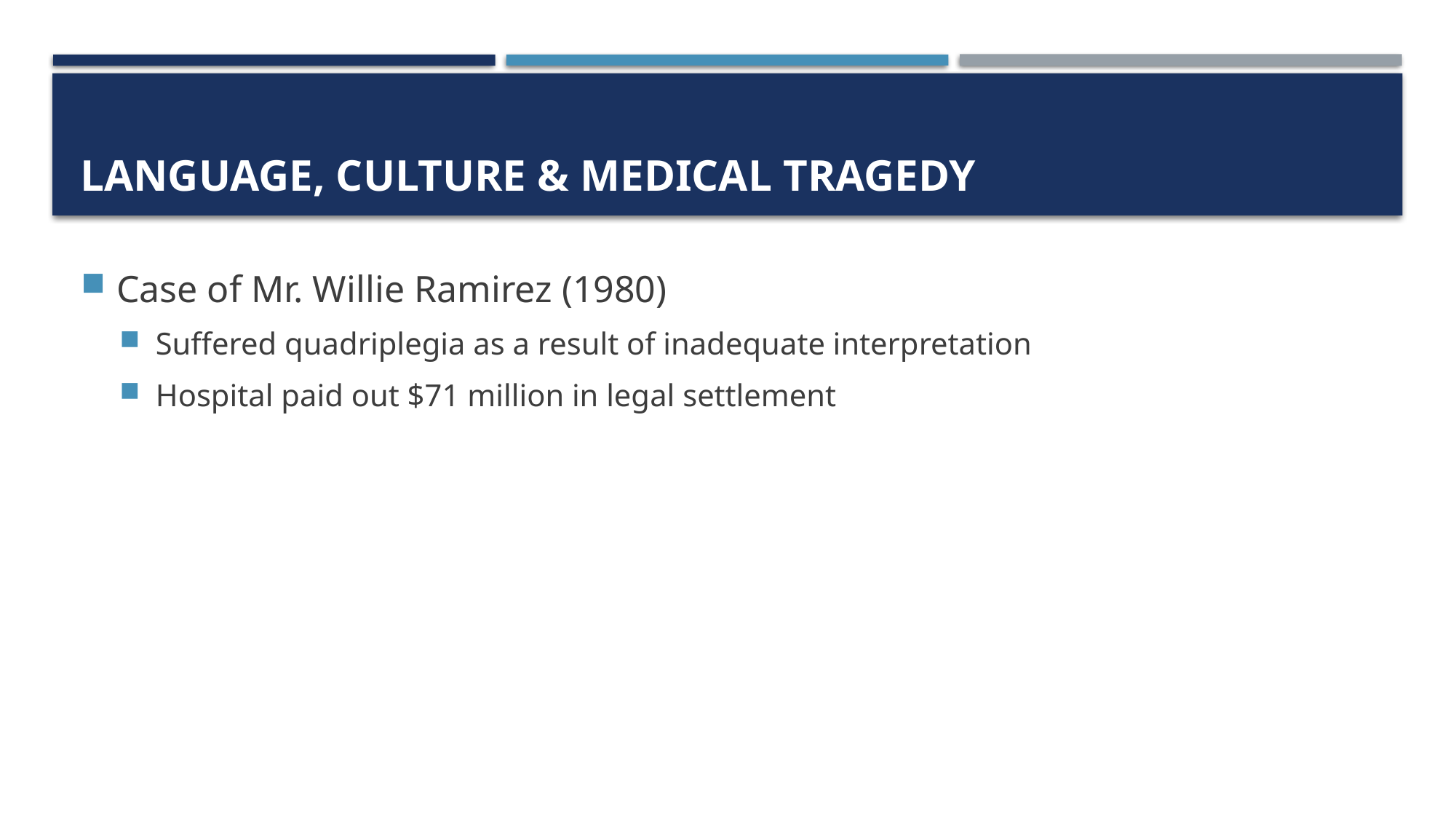

# Language, Culture & Medical Tragedy
Case of Mr. Willie Ramirez (1980)
Suffered quadriplegia as a result of inadequate interpretation
Hospital paid out $71 million in legal settlement

## Slide 4
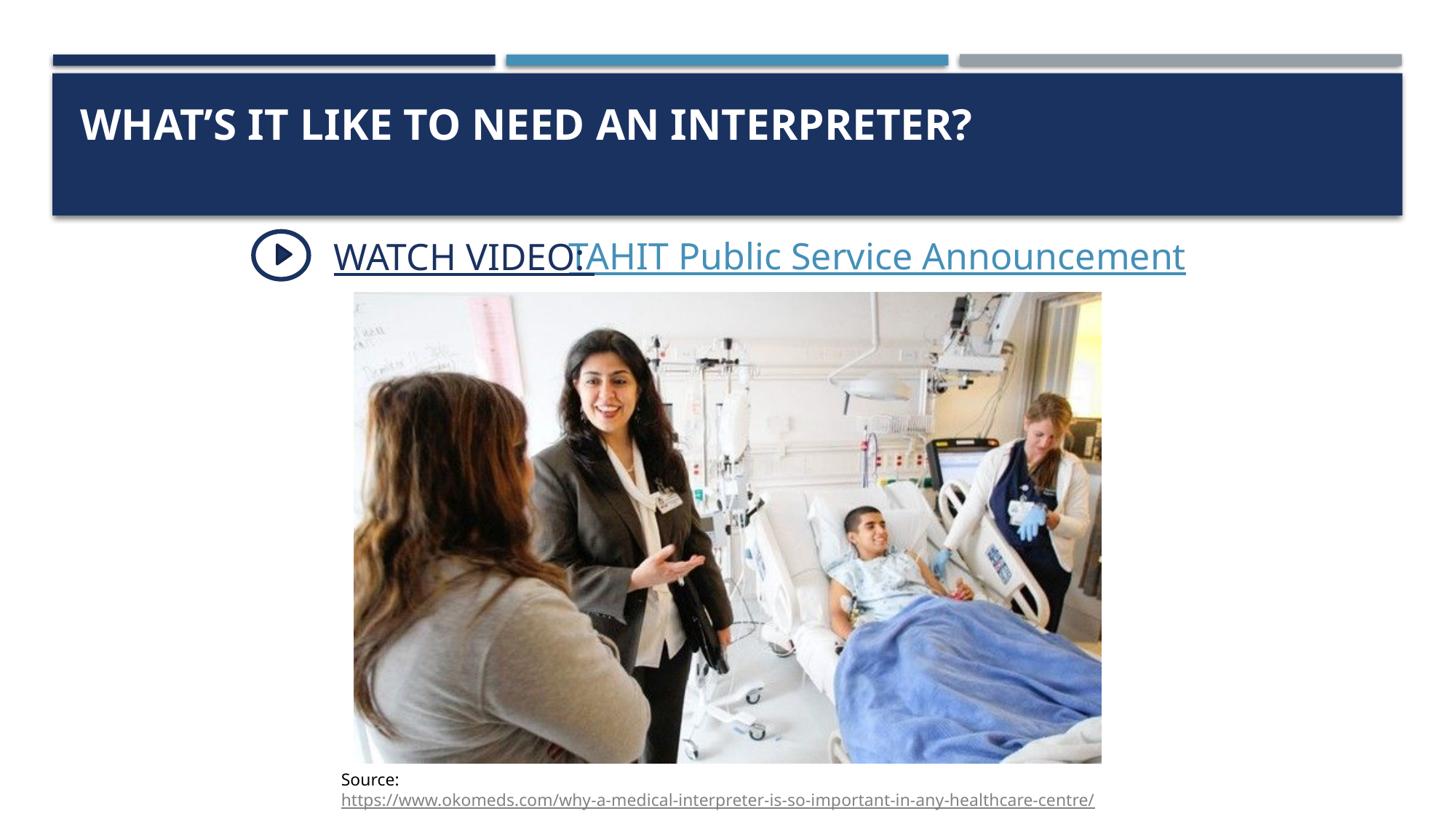

# What’s it like to need an interpreter?
TAHIT Public Service Announcement
WATCH VIDEO:
Source: https://www.okomeds.com/why-a-medical-interpreter-is-so-important-in-any-healthcare-centre/

## Slide 5
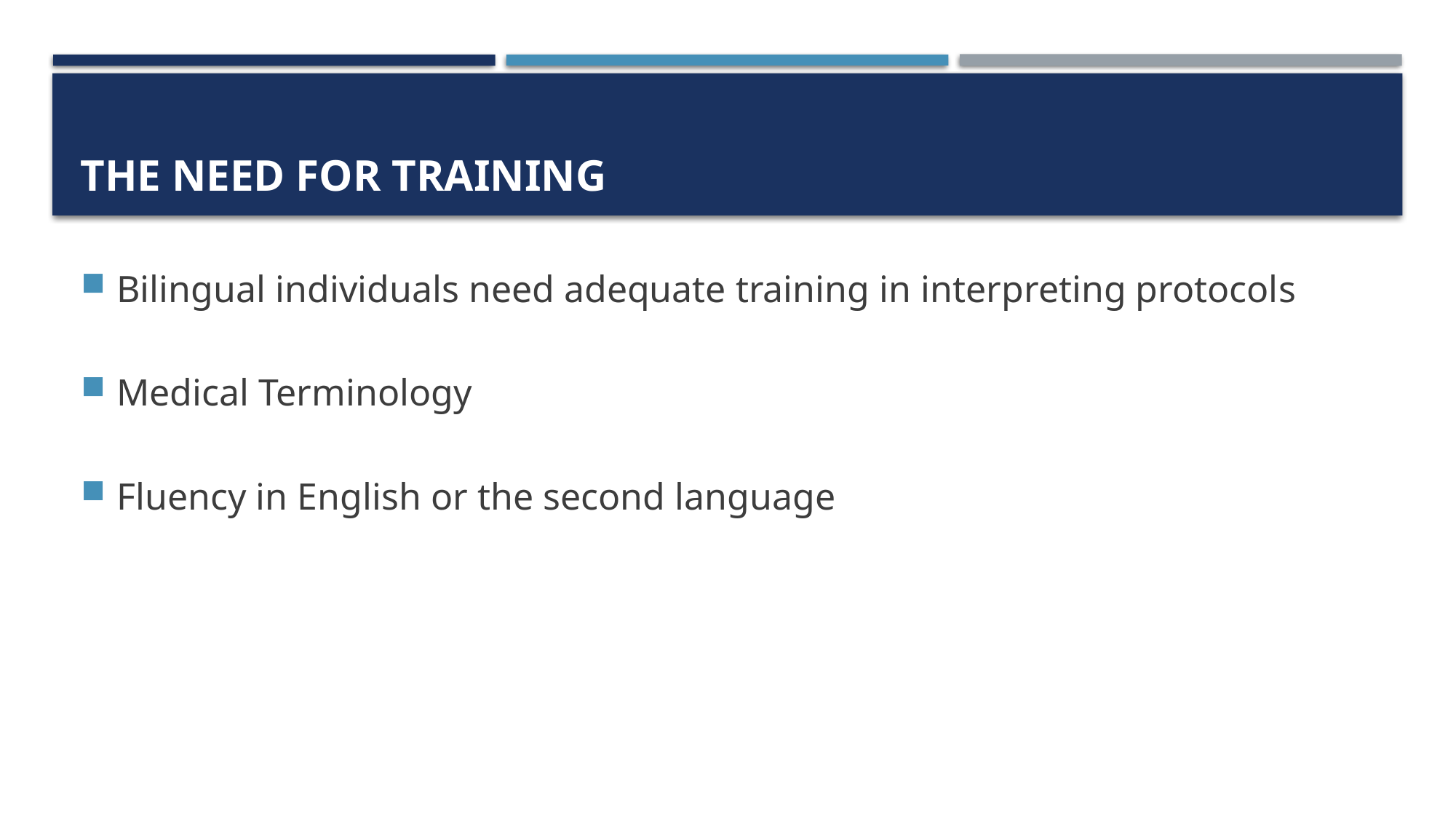

# The need for training
Bilingual individuals need adequate training in interpreting protocols
Medical Terminology
Fluency in English or the second language

## Slide 6
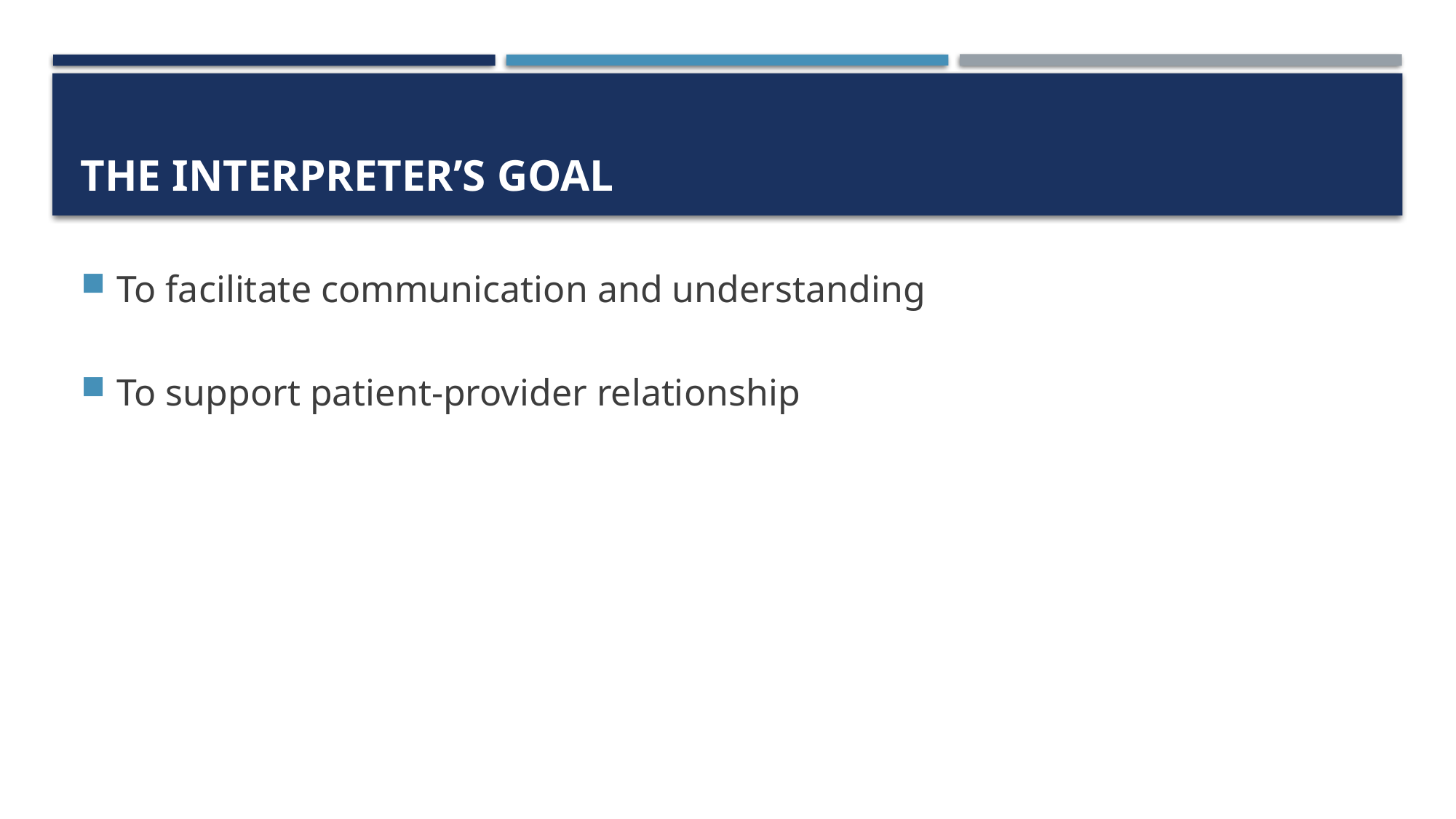

# The Interpreter’s Goal
To facilitate communication and understanding
To support patient-provider relationship

## Slide 7
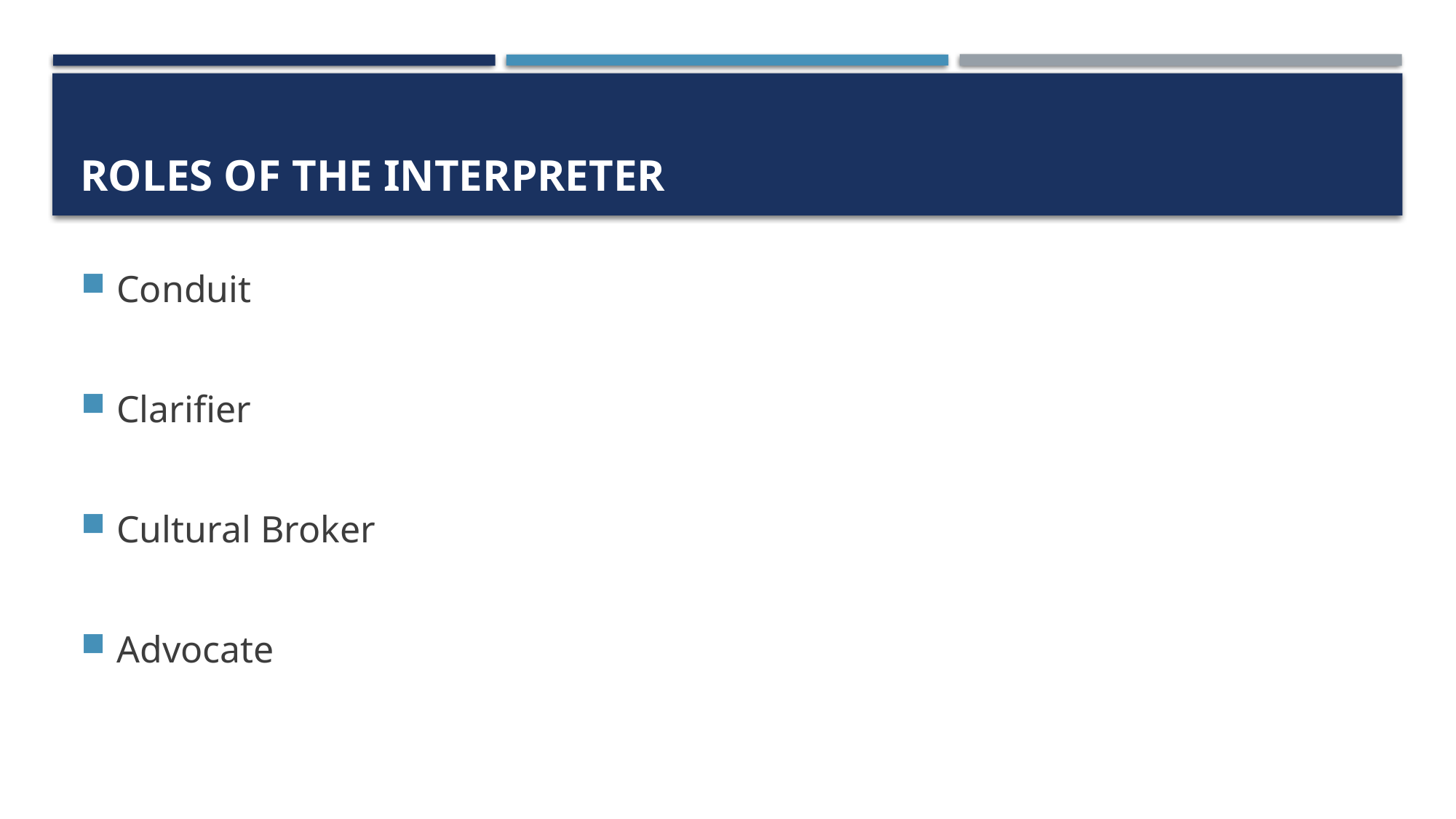

# Roles of the Interpreter
Conduit
Clarifier
Cultural Broker
Advocate

## Slide 8
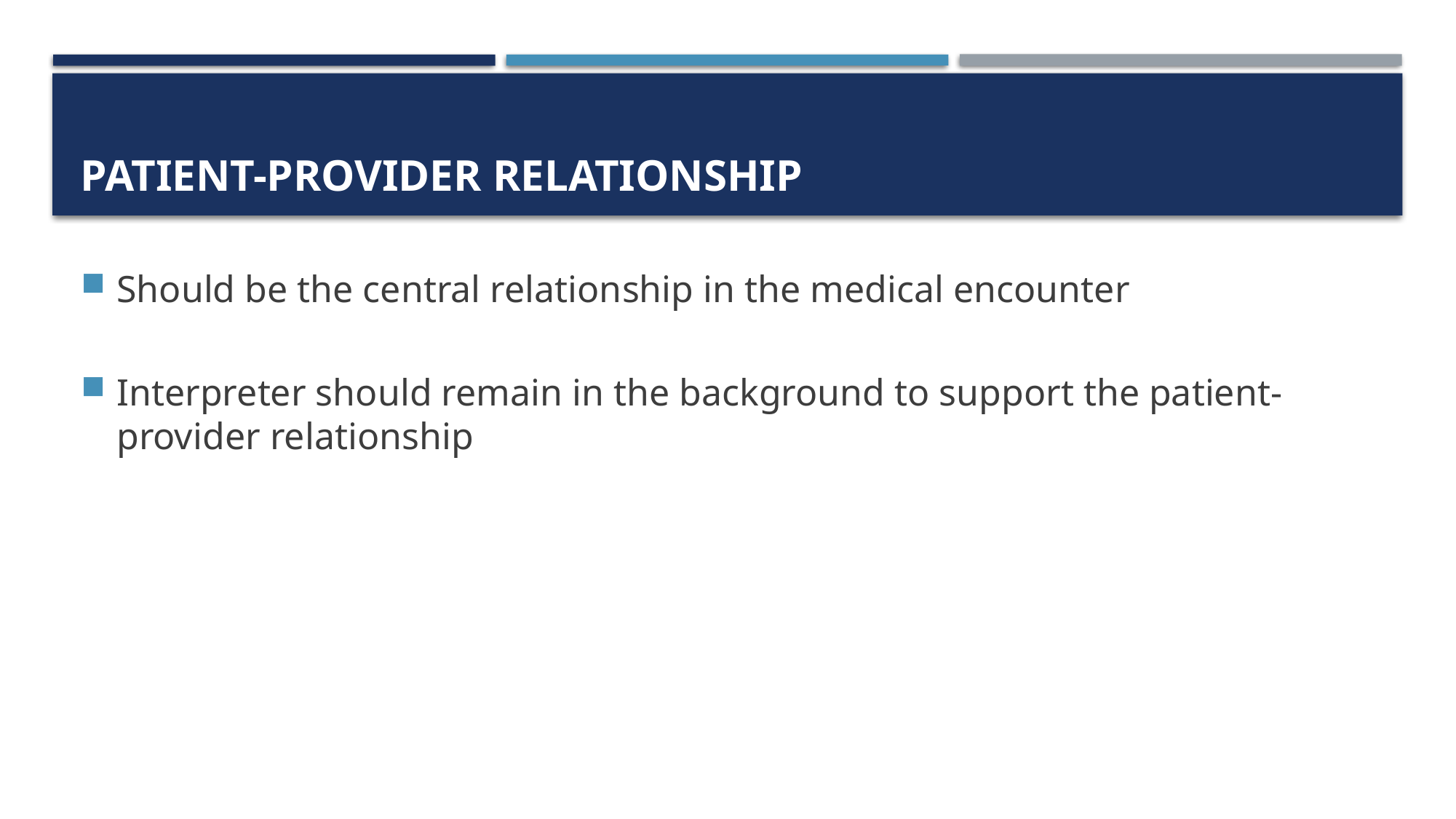

# Patient-Provider Relationship
Should be the central relationship in the medical encounter
Interpreter should remain in the background to support the patient-provider relationship

## Slide 9
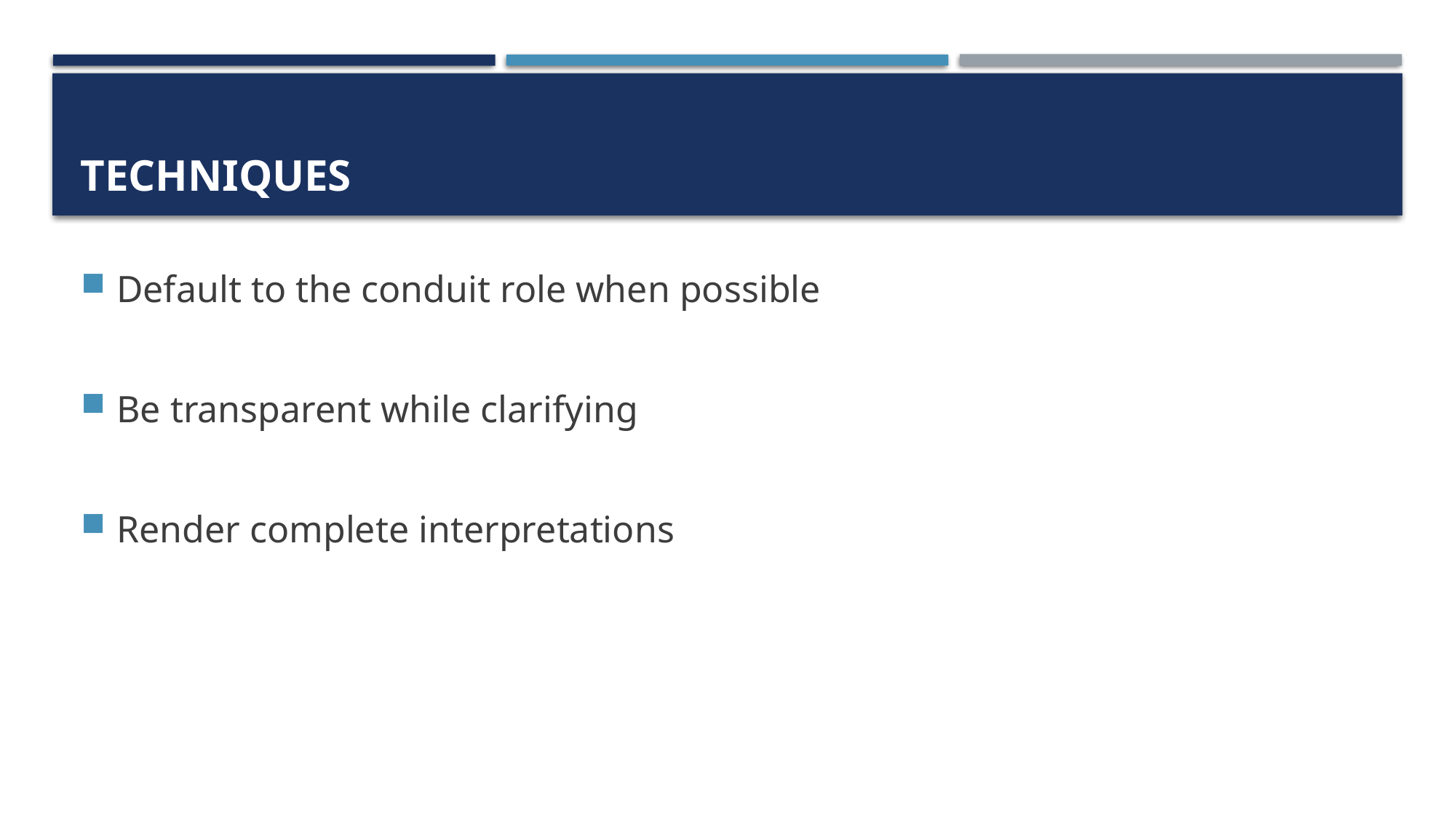

# Techniques
Default to the conduit role when possible
Be transparent while clarifying
Render complete interpretations

## Slide 10
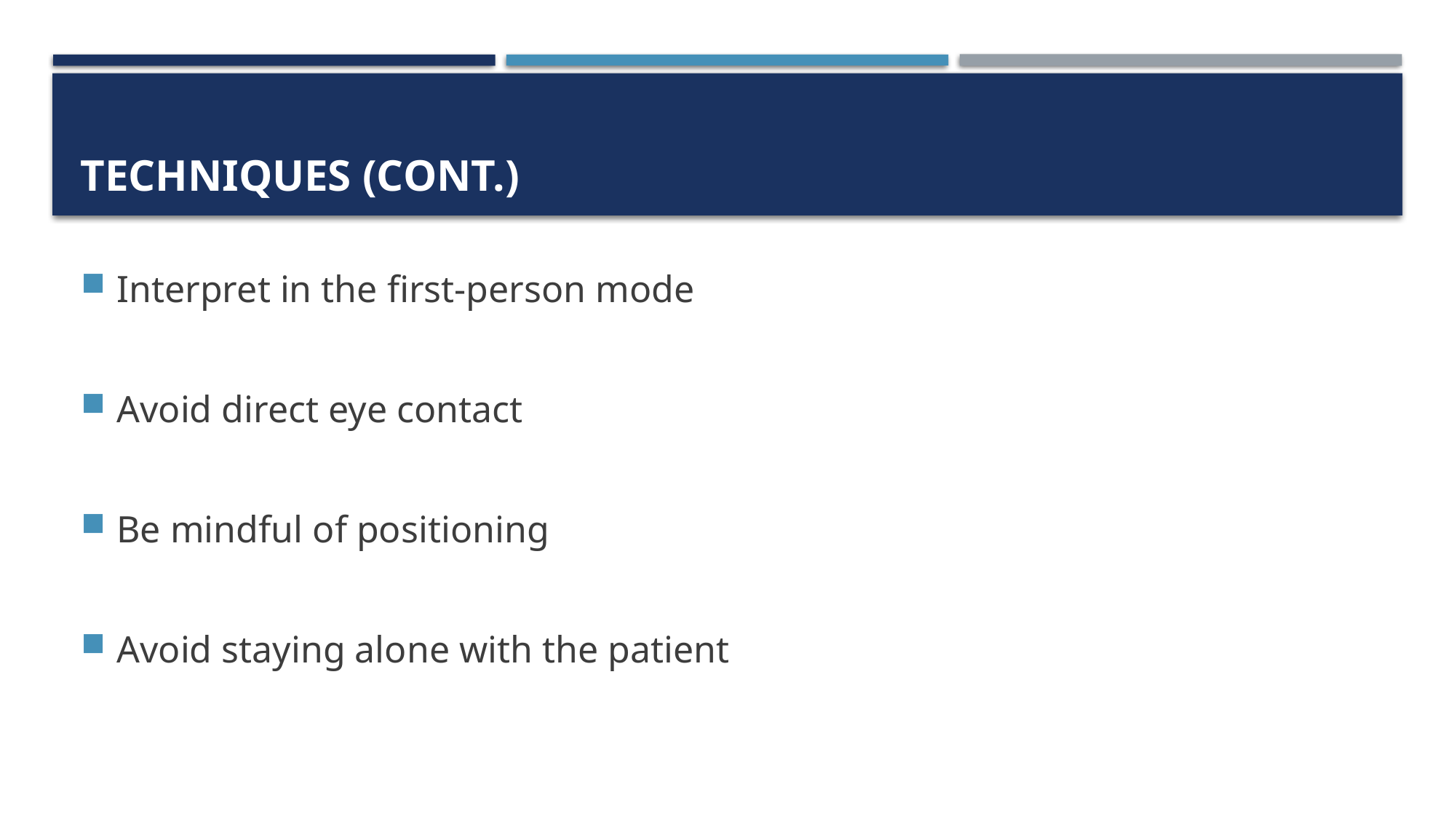

# Techniques (cont.)
Interpret in the first-person mode
Avoid direct eye contact
Be mindful of positioning
Avoid staying alone with the patient

## Slide 11
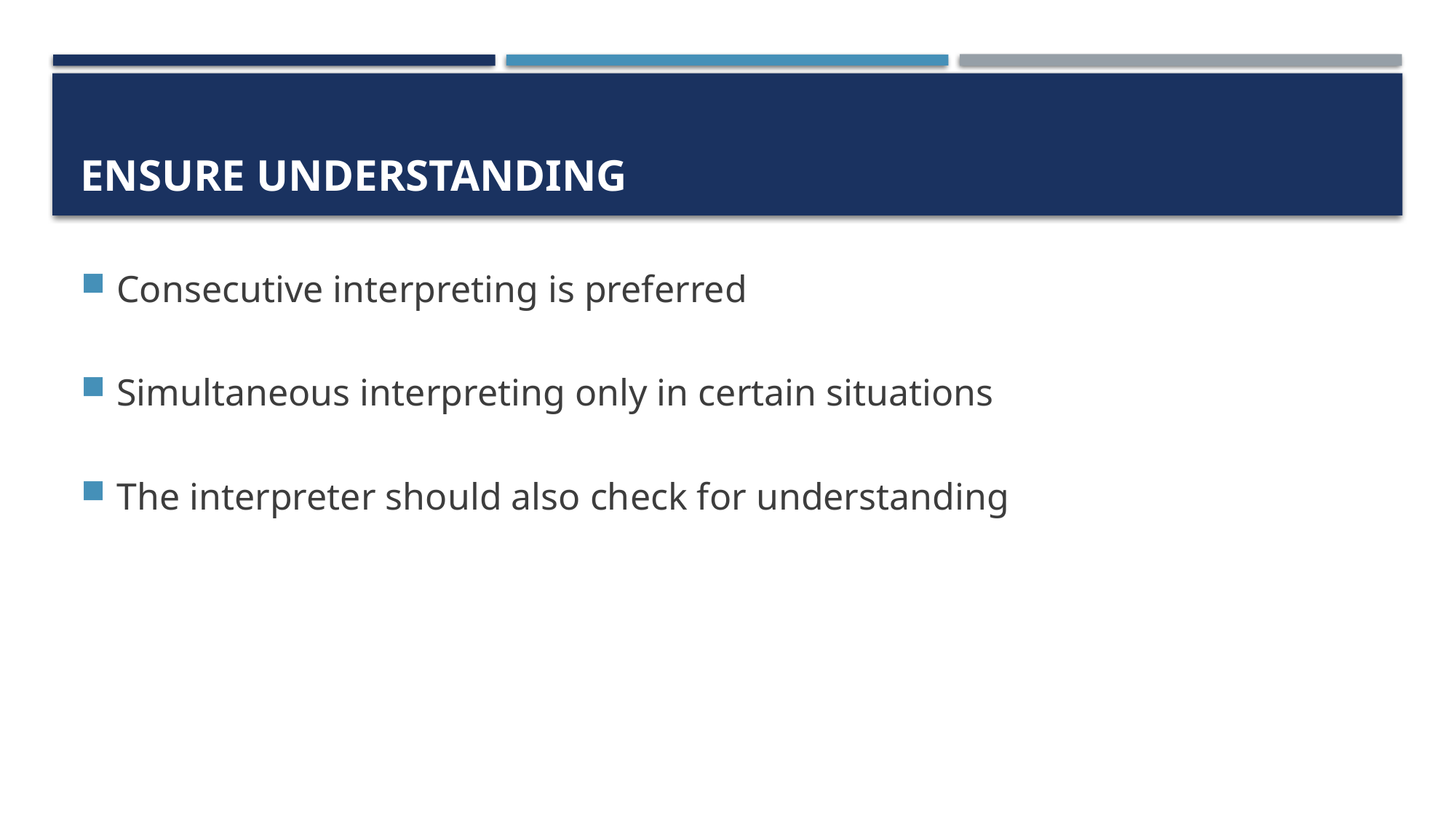

# Ensure Understanding
Consecutive interpreting is preferred
Simultaneous interpreting only in certain situations
The interpreter should also check for understanding

## Slide 12
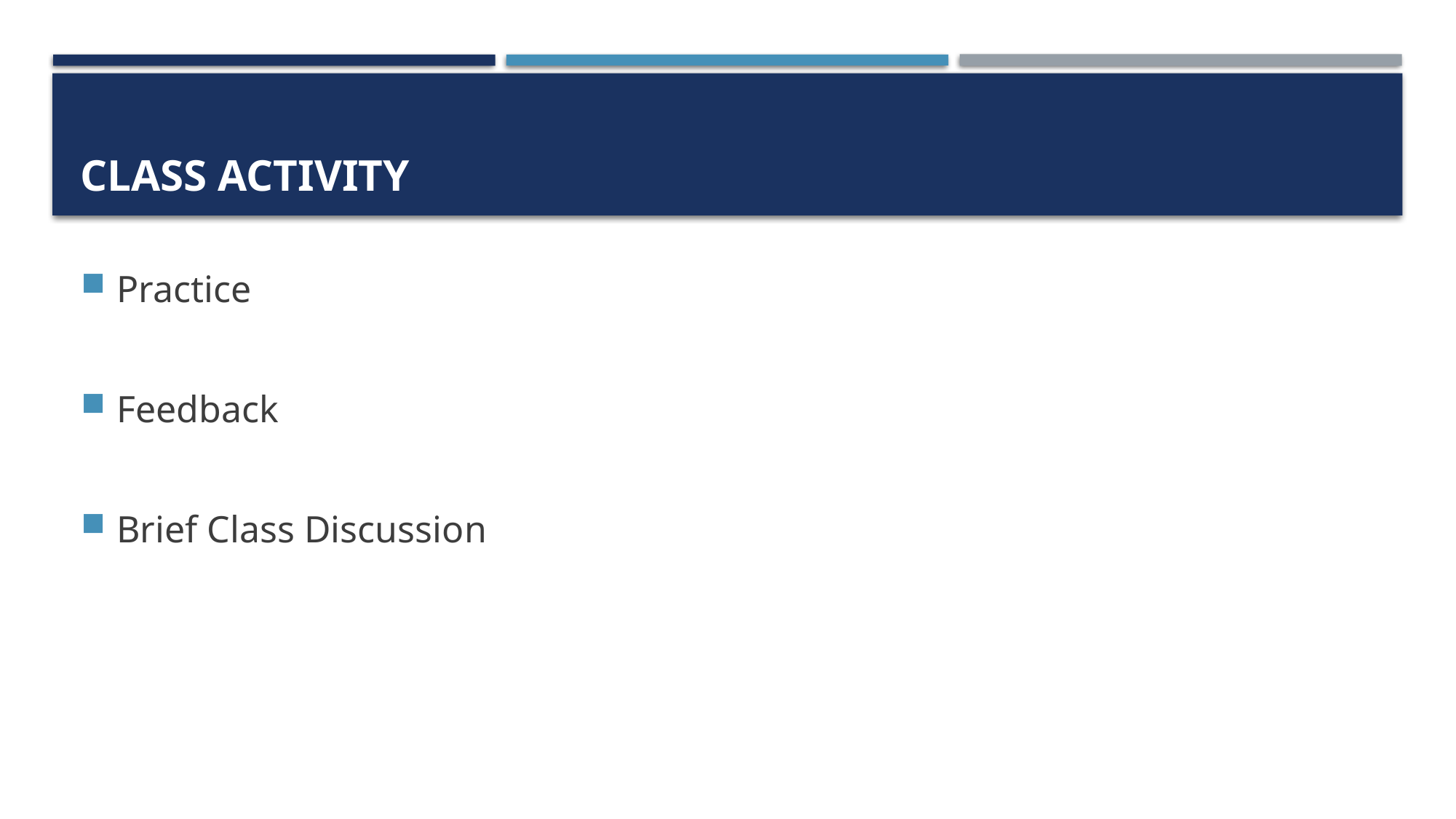

# Class Activity
Practice
Feedback
Brief Class Discussion

## Slide 13
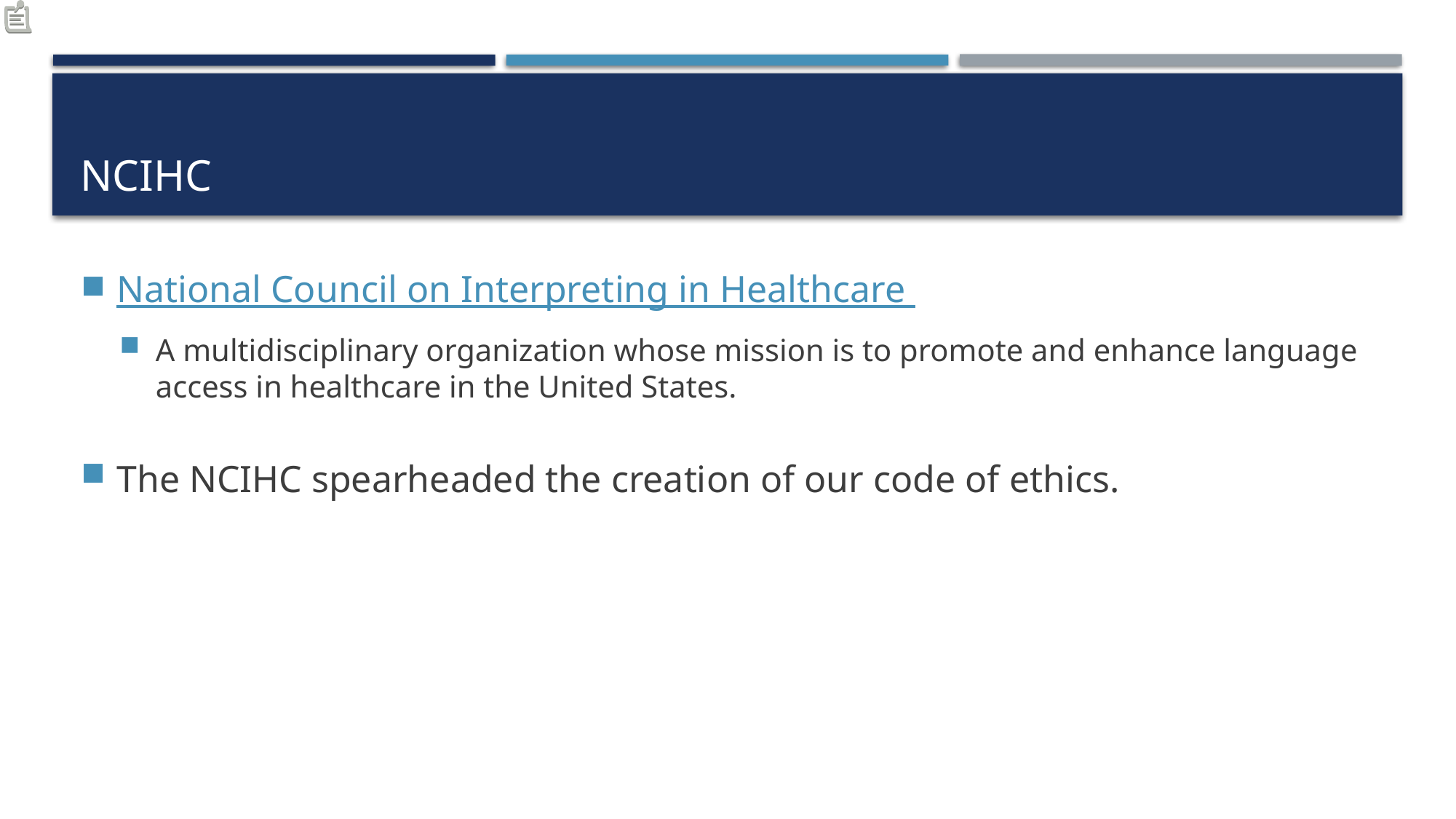

# NCIHC
National Council on Interpreting in Healthcare
A multidisciplinary organization whose mission is to promote and enhance language access in healthcare in the United States.
The NCIHC spearheaded the creation of our code of ethics.

## Slide 14
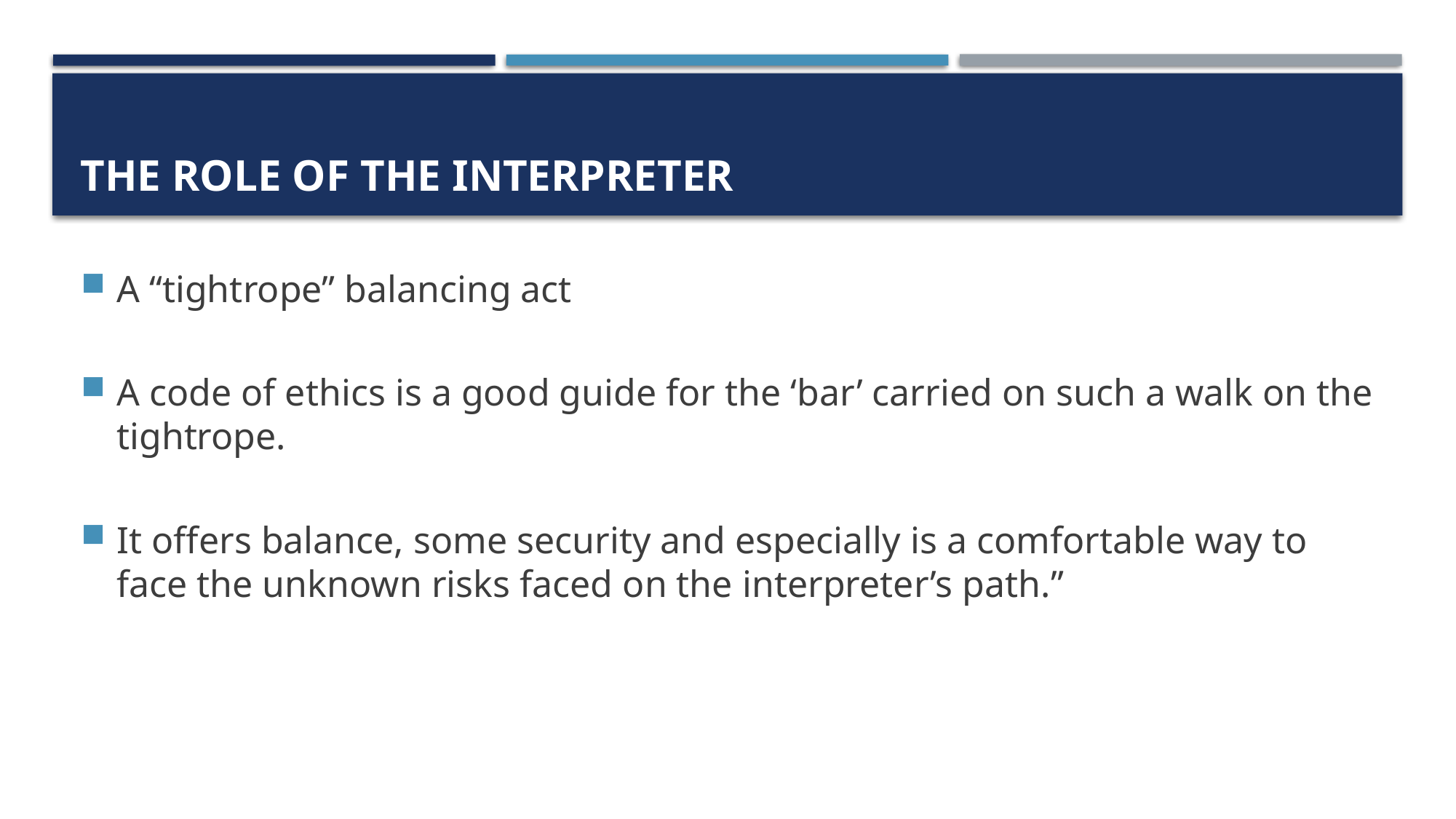

# The Role of the Interpreter
A “tightrope” balancing act
A code of ethics is a good guide for the ‘bar’ carried on such a walk on the tightrope.
It offers balance, some security and especially is a comfortable way to face the unknown risks faced on the interpreter’s path.”

## Slide 15
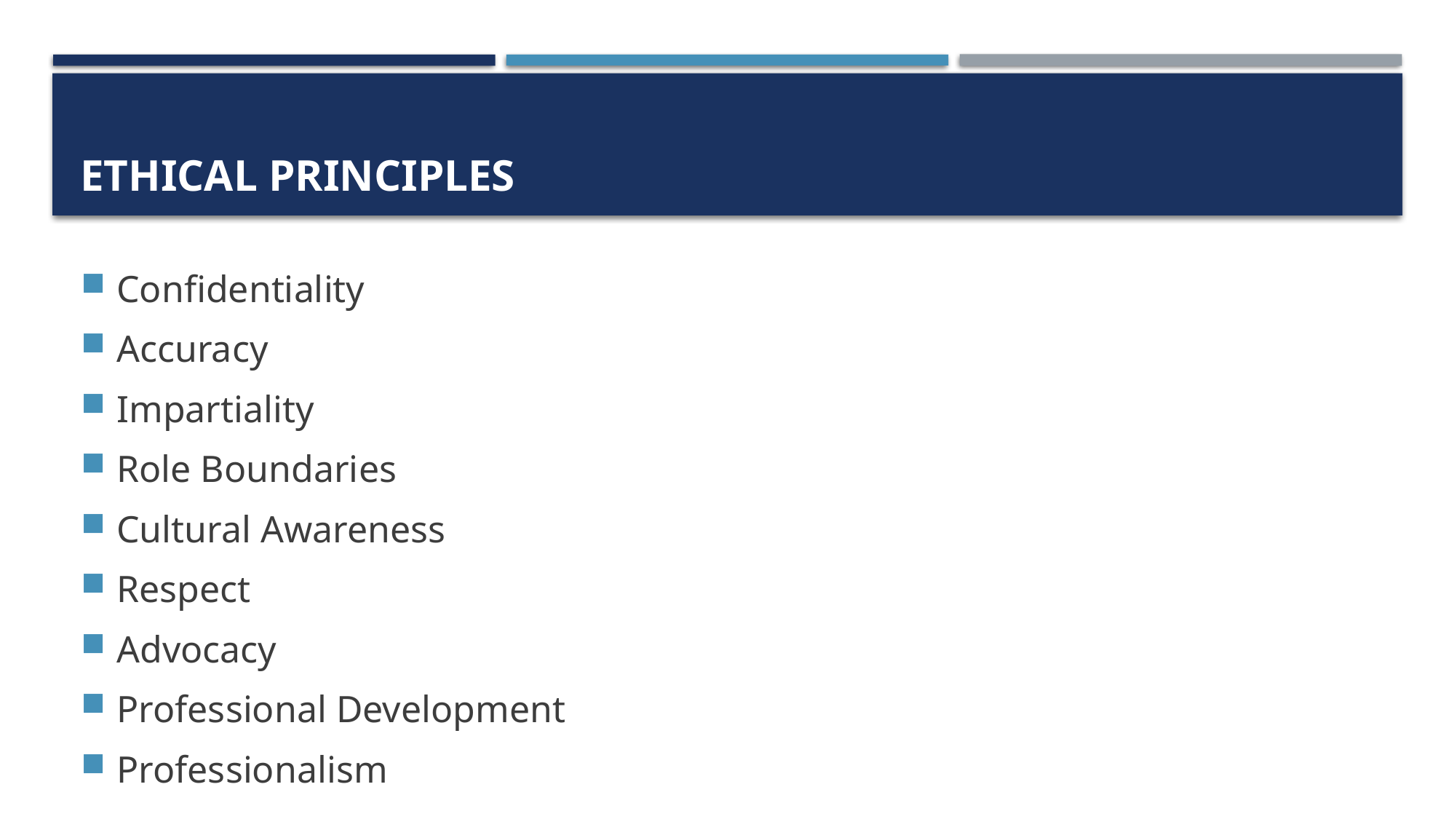

# Ethical Principles
Confidentiality
Accuracy
Impartiality
Role Boundaries
Cultural Awareness
Respect
Advocacy
Professional Development
Professionalism

## Slide 16
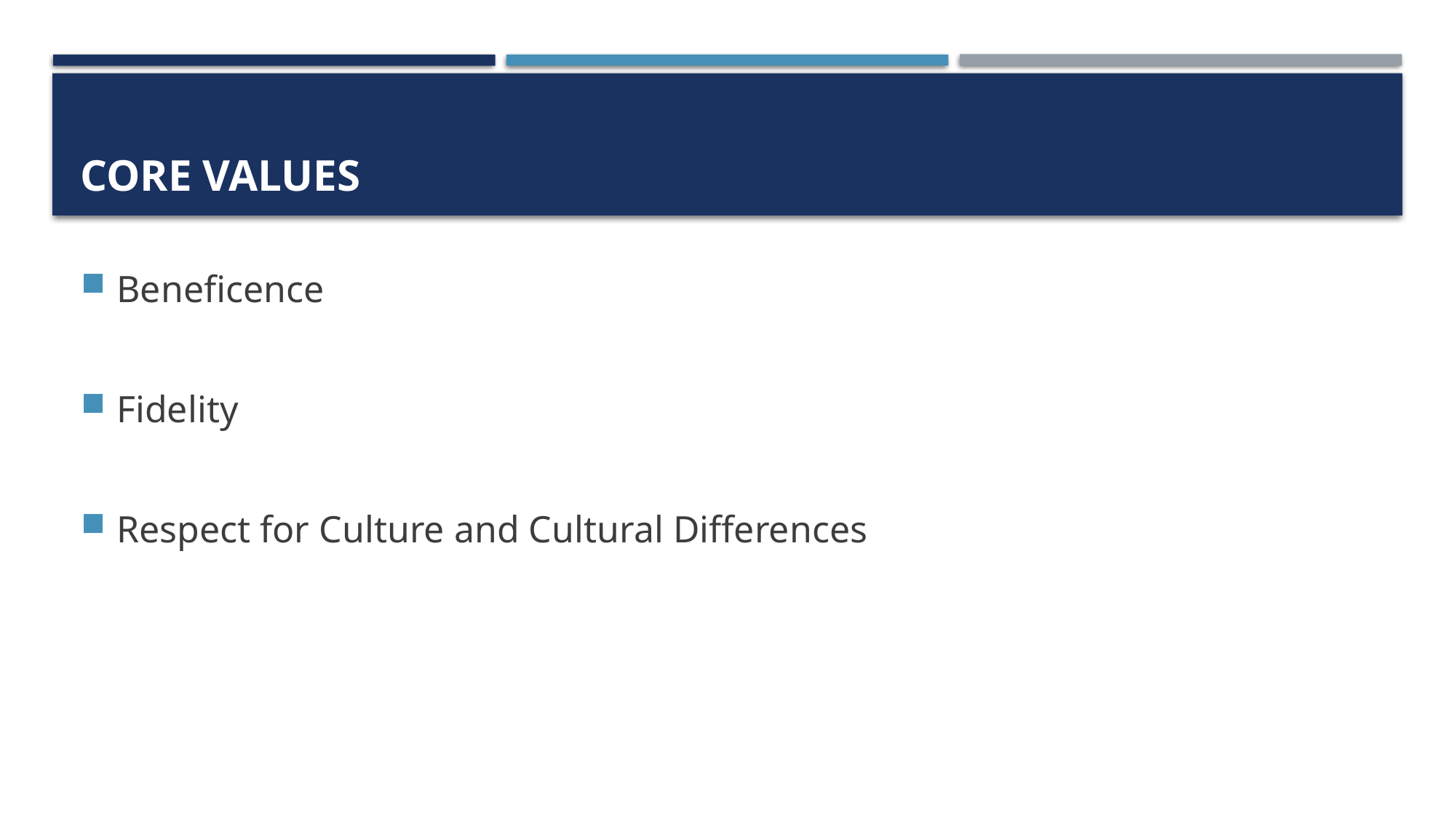

# Core Values
Beneficence
Fidelity
Respect for Culture and Cultural Differences

## Slide 17
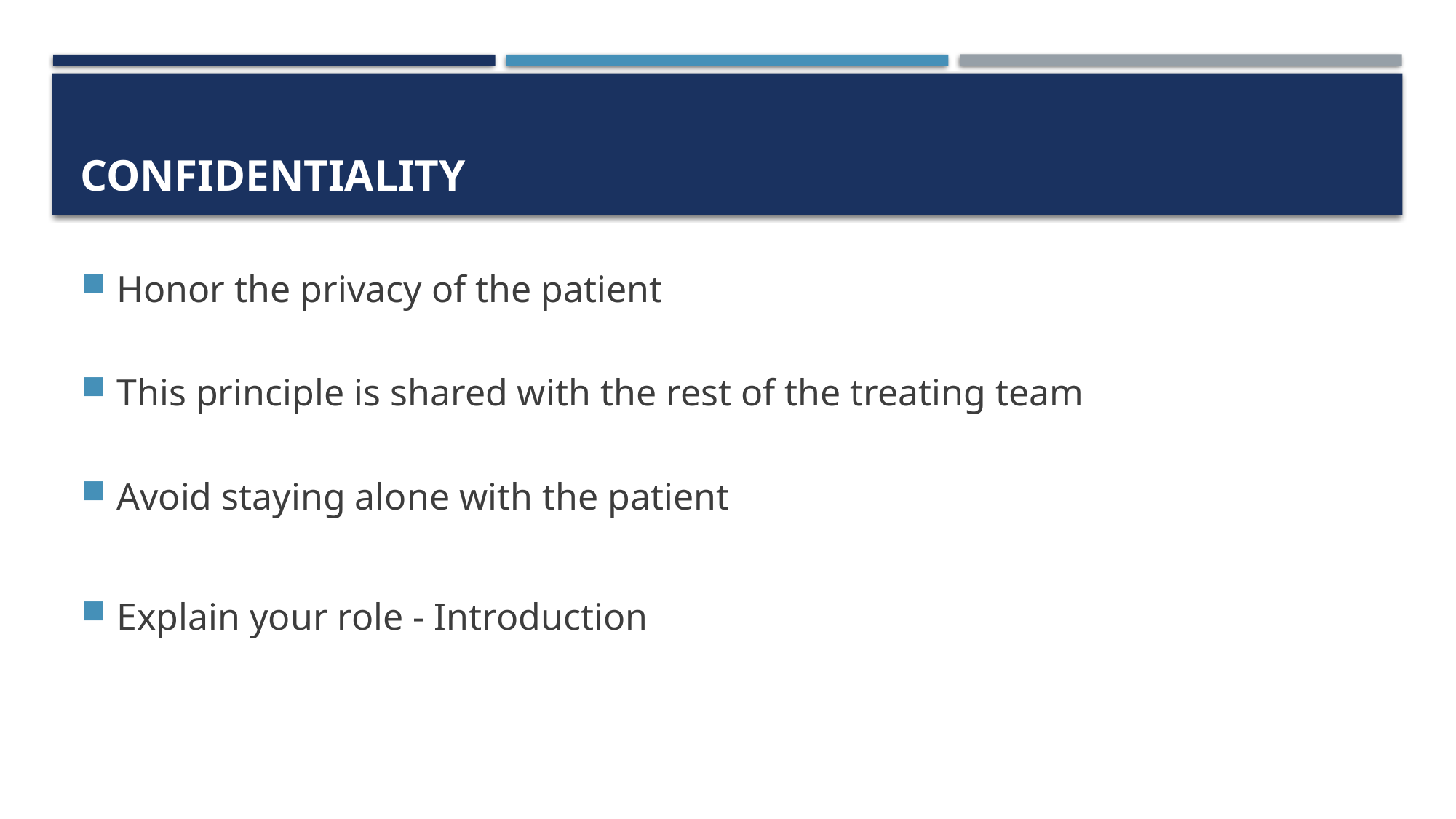

# Confidentiality
Honor the privacy of the patient
This principle is shared with the rest of the treating team
Avoid staying alone with the patient
Explain your role - Introduction

## Slide 18
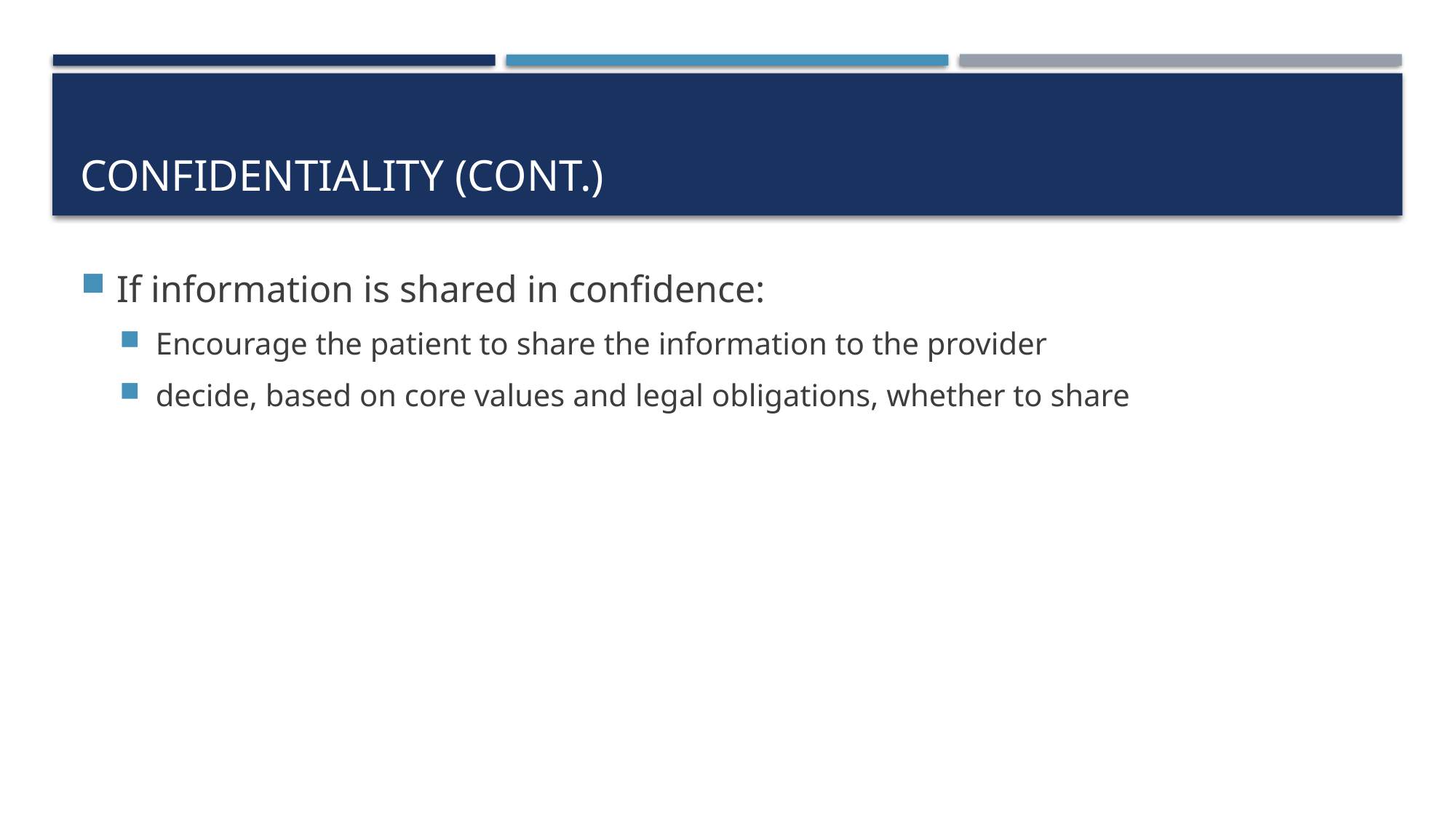

# Confidentiality (cont.)
If information is shared in confidence:
Encourage the patient to share the information to the provider
decide, based on core values and legal obligations, whether to share

## Slide 19
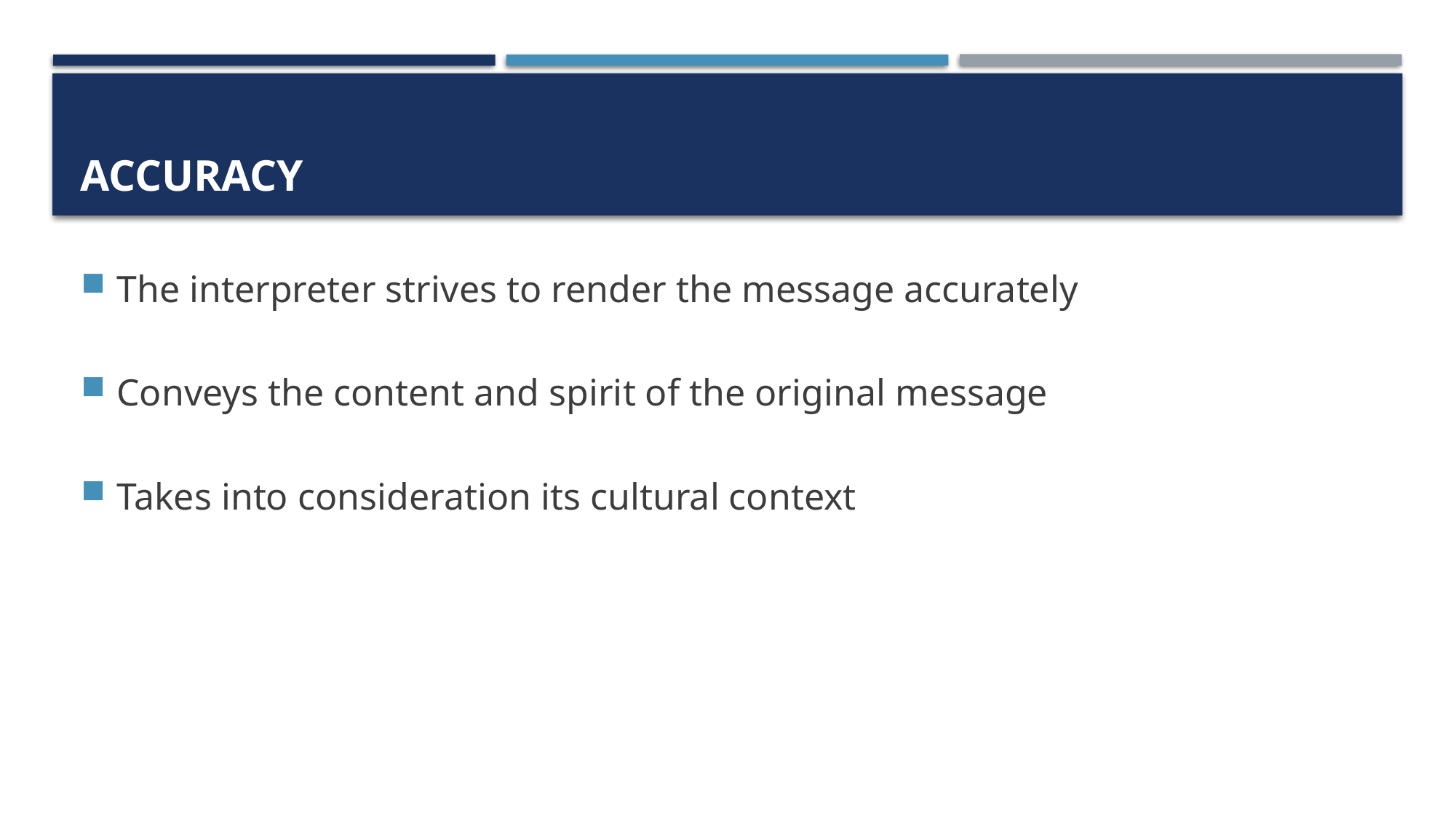

# Accuracy
The interpreter strives to render the message accurately
Conveys the content and spirit of the original message
Takes into consideration its cultural context

## Slide 20
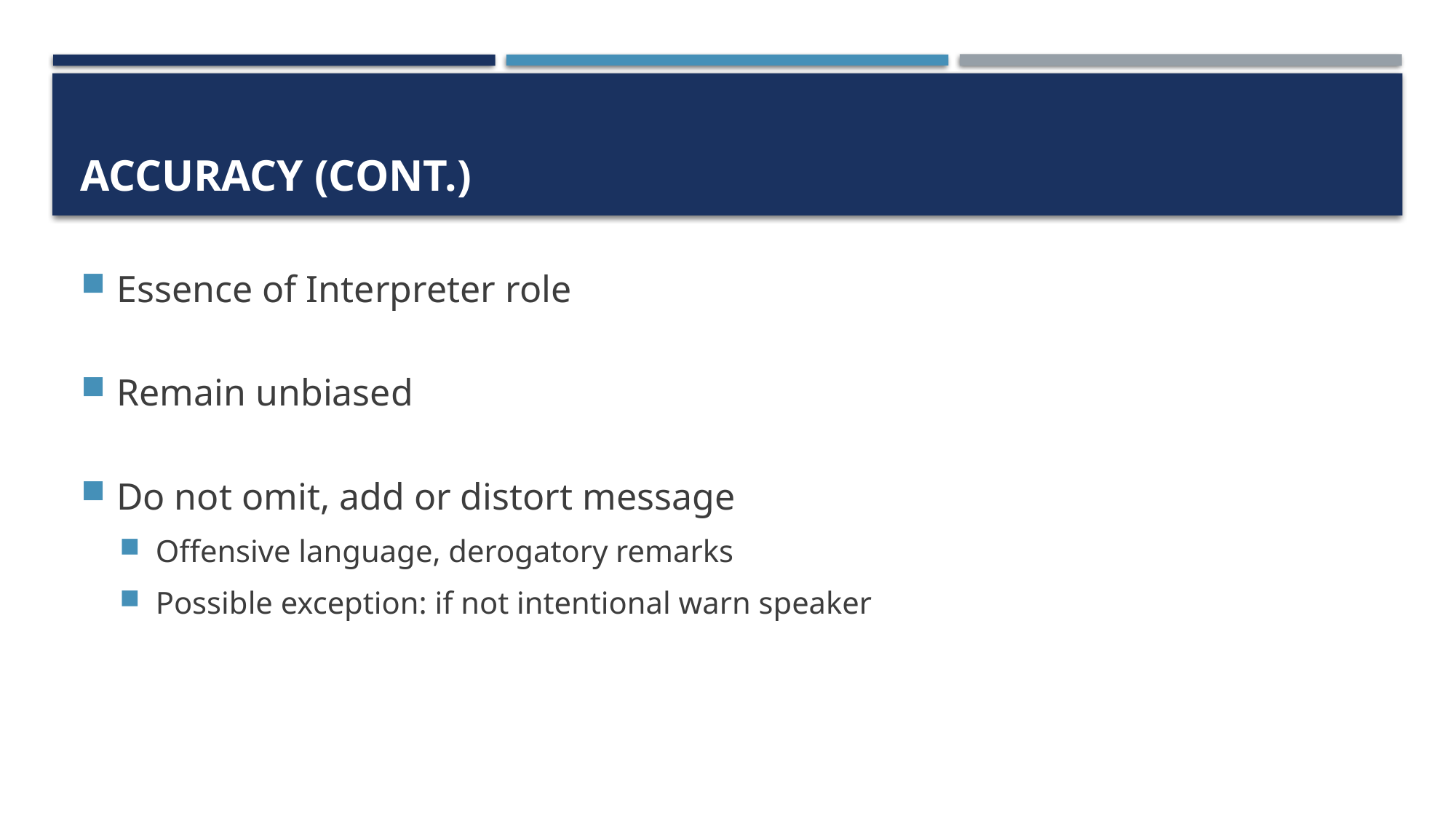

# Accuracy (cont.)
Essence of Interpreter role
Remain unbiased
Do not omit, add or distort message
Offensive language, derogatory remarks
Possible exception: if not intentional warn speaker

## Slide 21
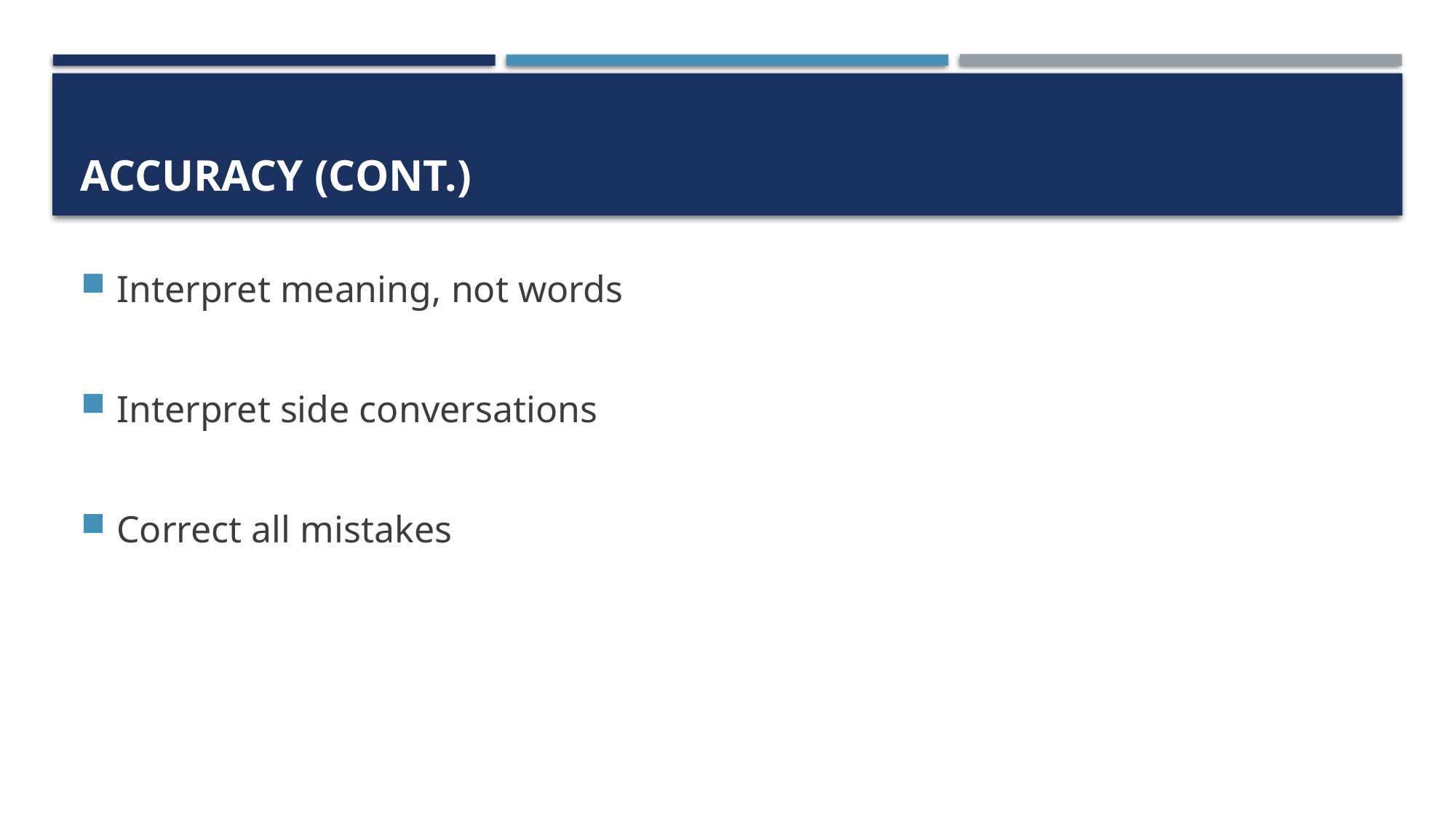

# Accuracy (cont.)
Interpret meaning, not words
Interpret side conversations
Correct all mistakes

## Slide 22
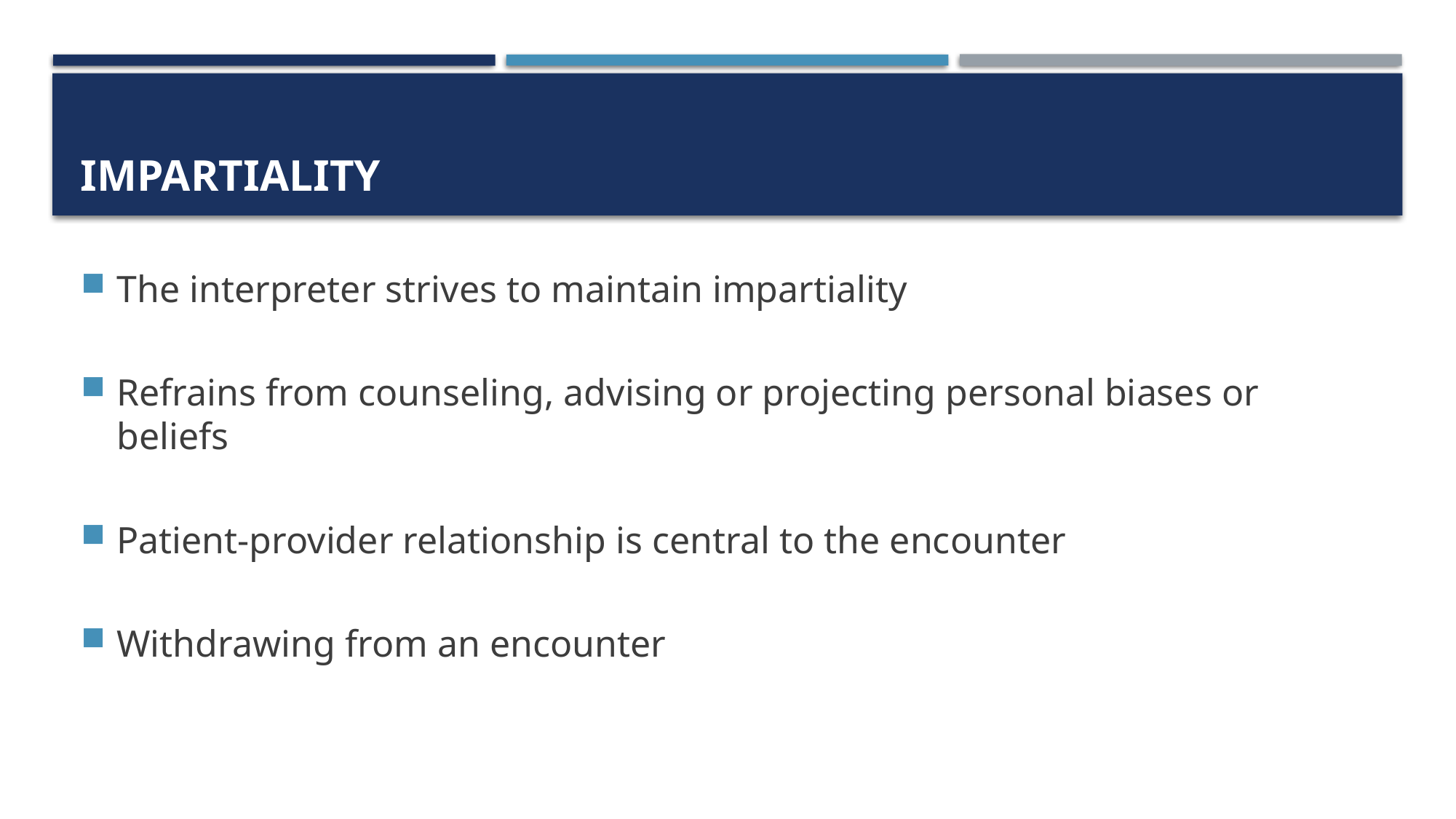

# Impartiality
The interpreter strives to maintain impartiality
Refrains from counseling, advising or projecting personal biases or beliefs
Patient-provider relationship is central to the encounter
Withdrawing from an encounter

## Slide 23
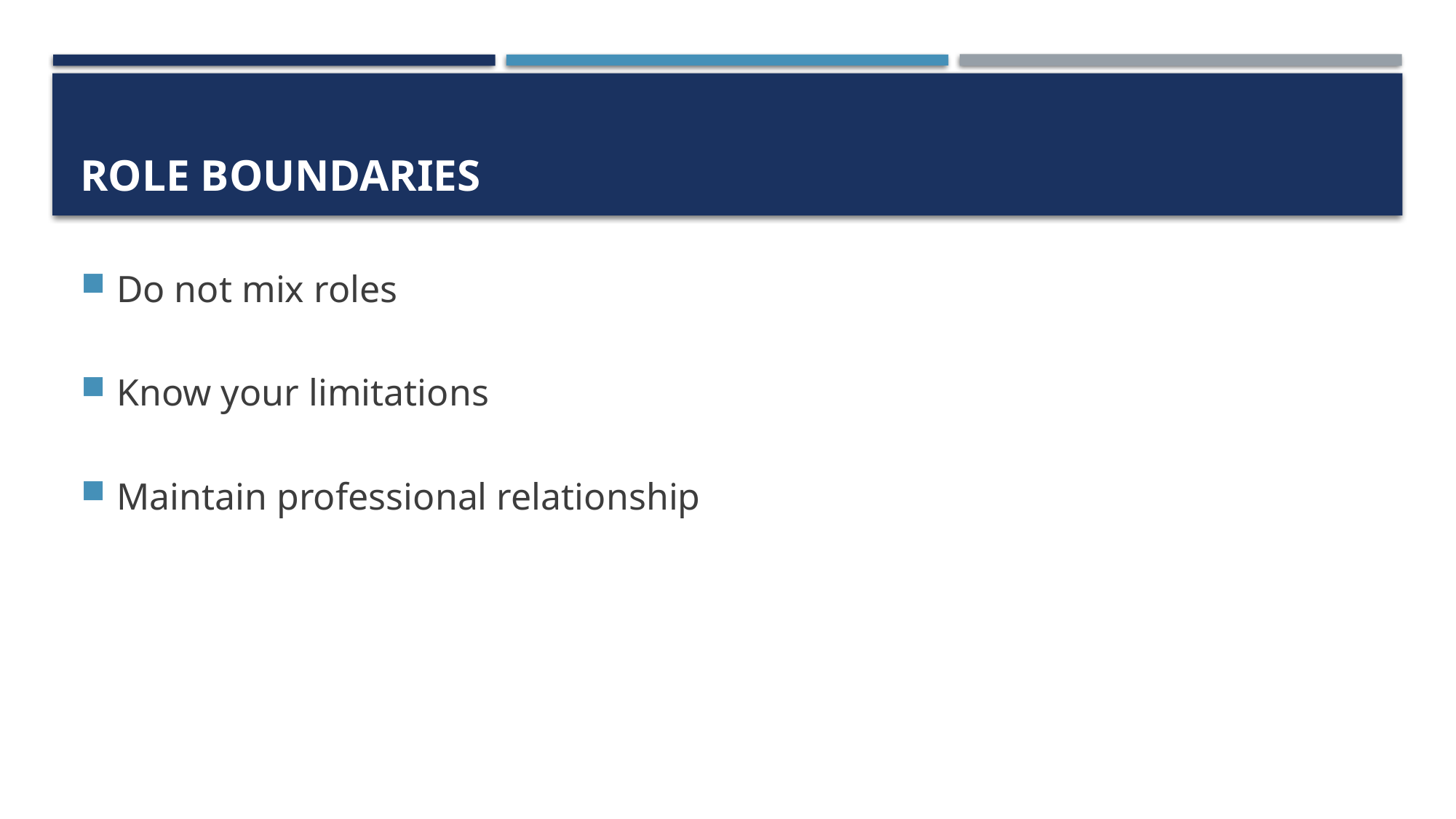

# Role Boundaries
Do not mix roles
Know your limitations
Maintain professional relationship

## Slide 24
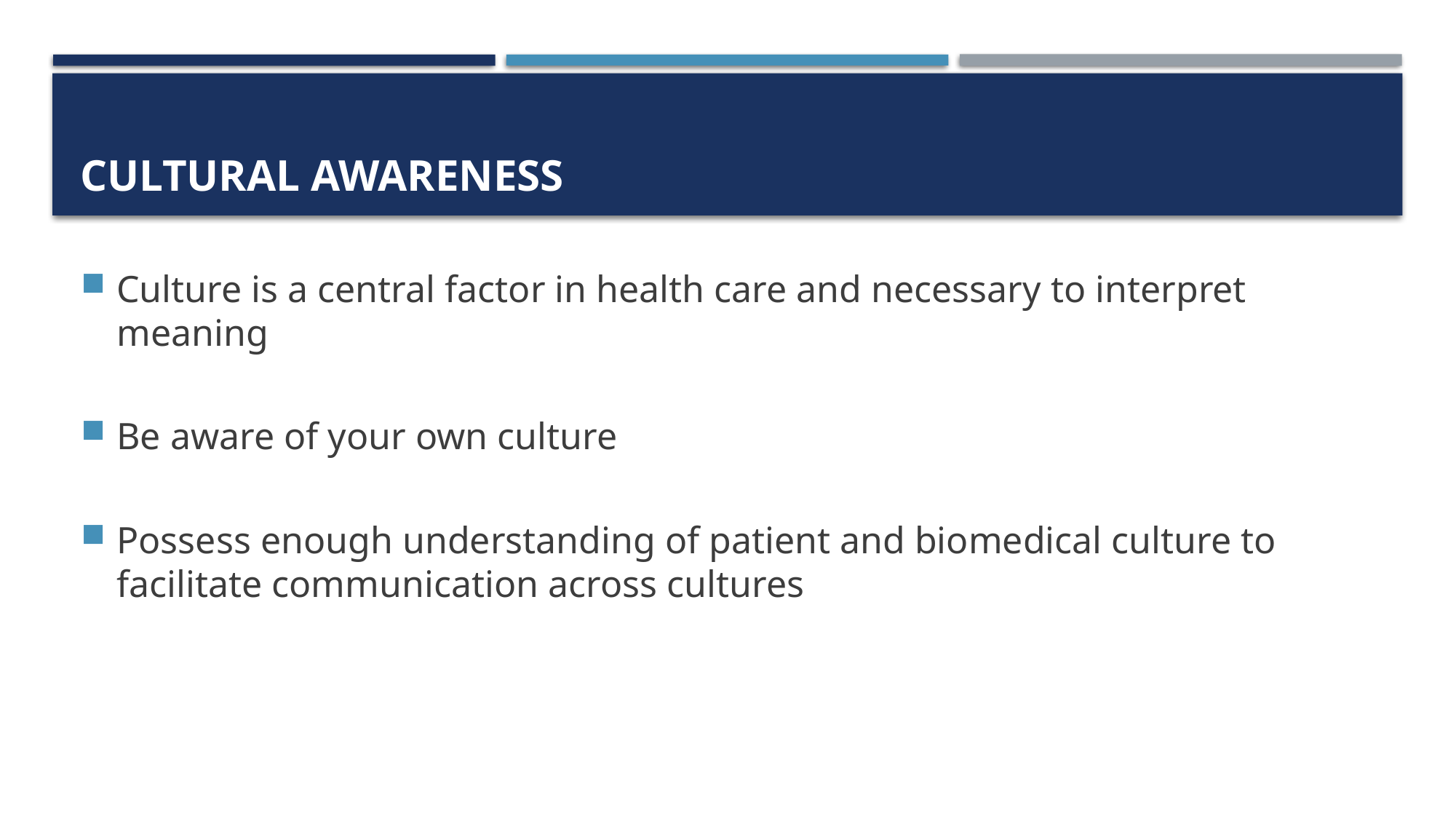

# Cultural Awareness
Culture is a central factor in health care and necessary to interpret meaning
Be aware of your own culture
Possess enough understanding of patient and biomedical culture to facilitate communication across cultures

## Slide 25
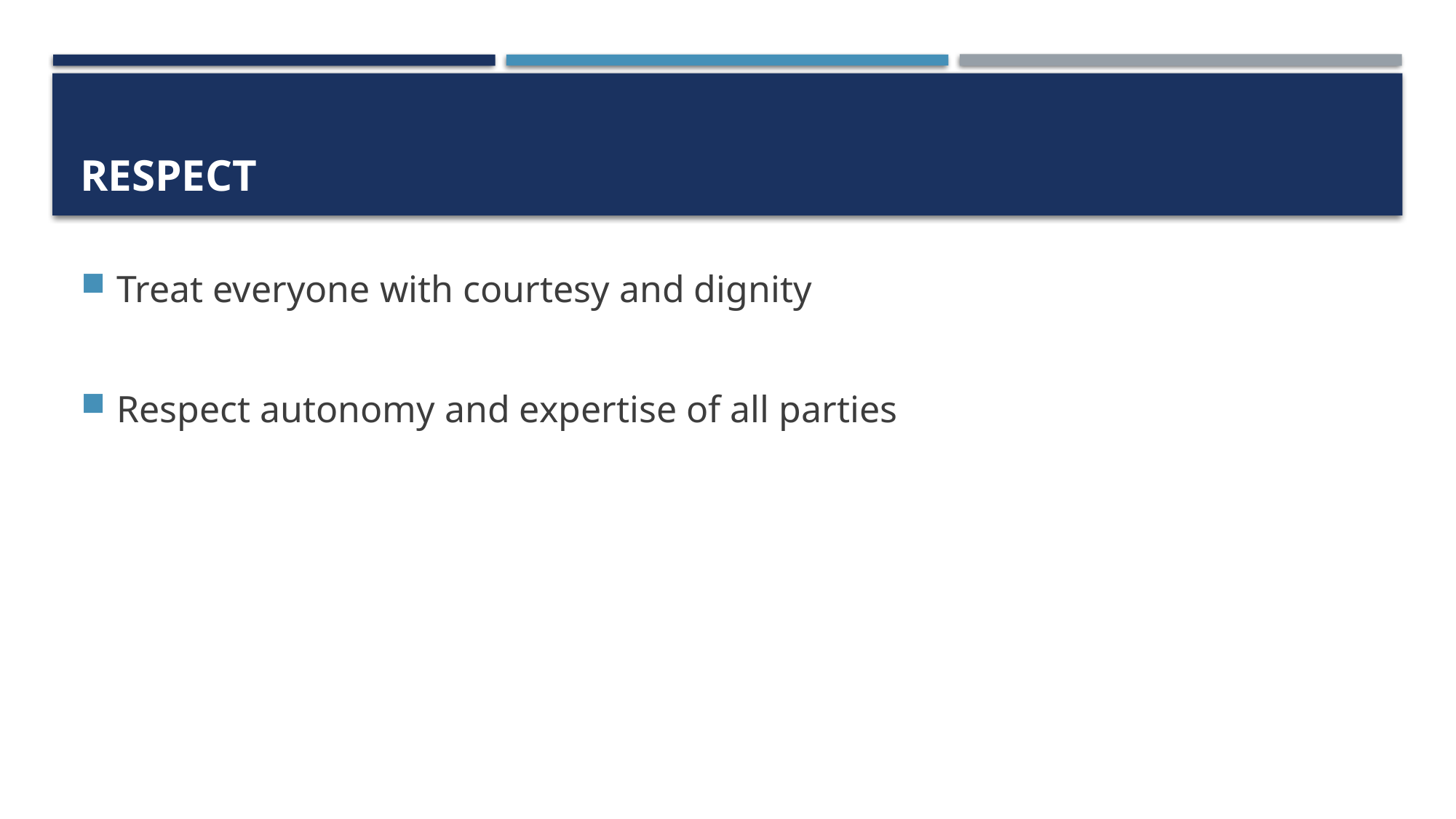

# Respect
Treat everyone with courtesy and dignity
Respect autonomy and expertise of all parties

## Slide 26
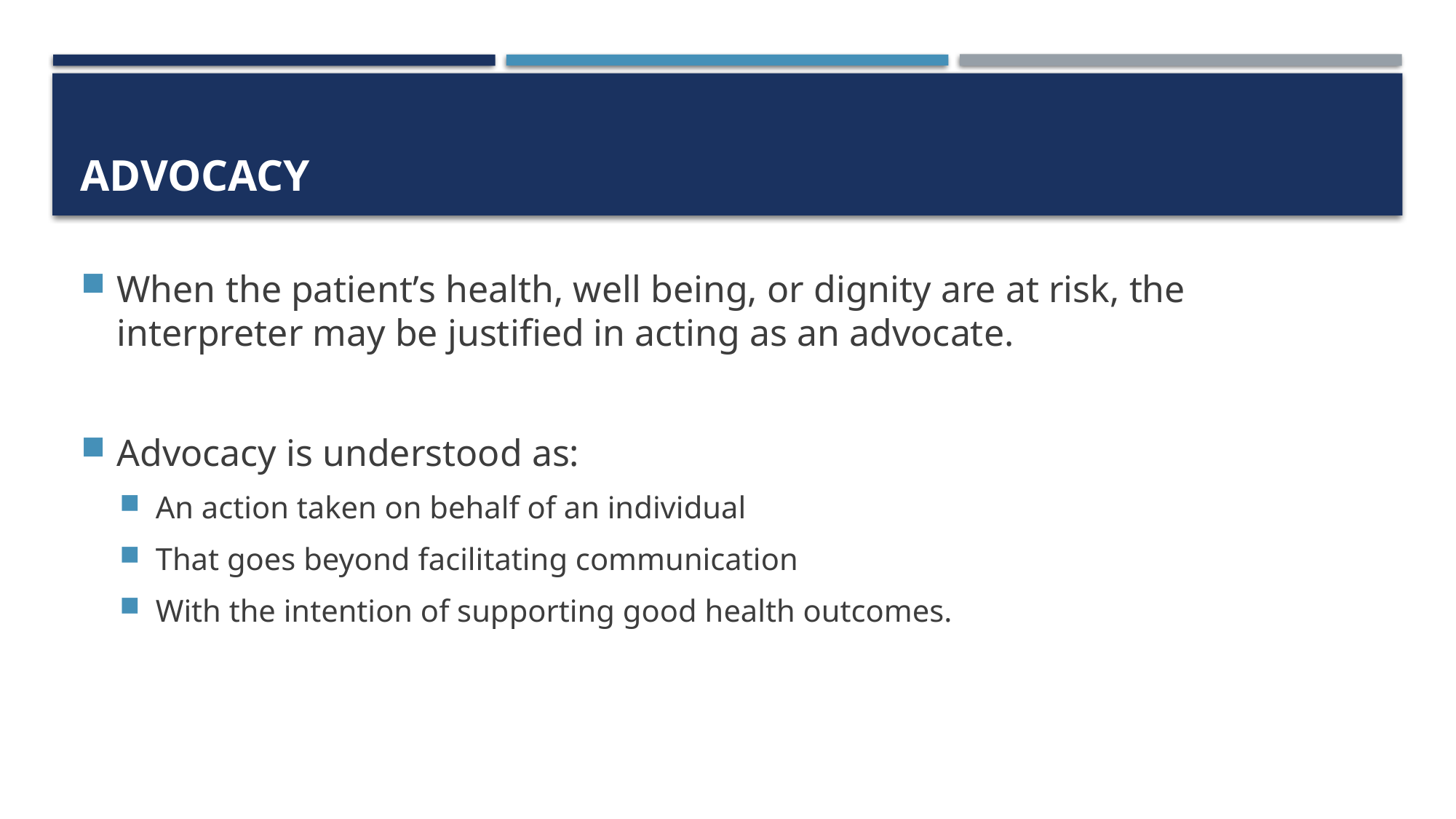

# Advocacy
When the patient’s health, well being, or dignity are at risk, the interpreter may be justified in acting as an advocate.
Advocacy is understood as:
An action taken on behalf of an individual
That goes beyond facilitating communication
With the intention of supporting good health outcomes.

## Slide 27
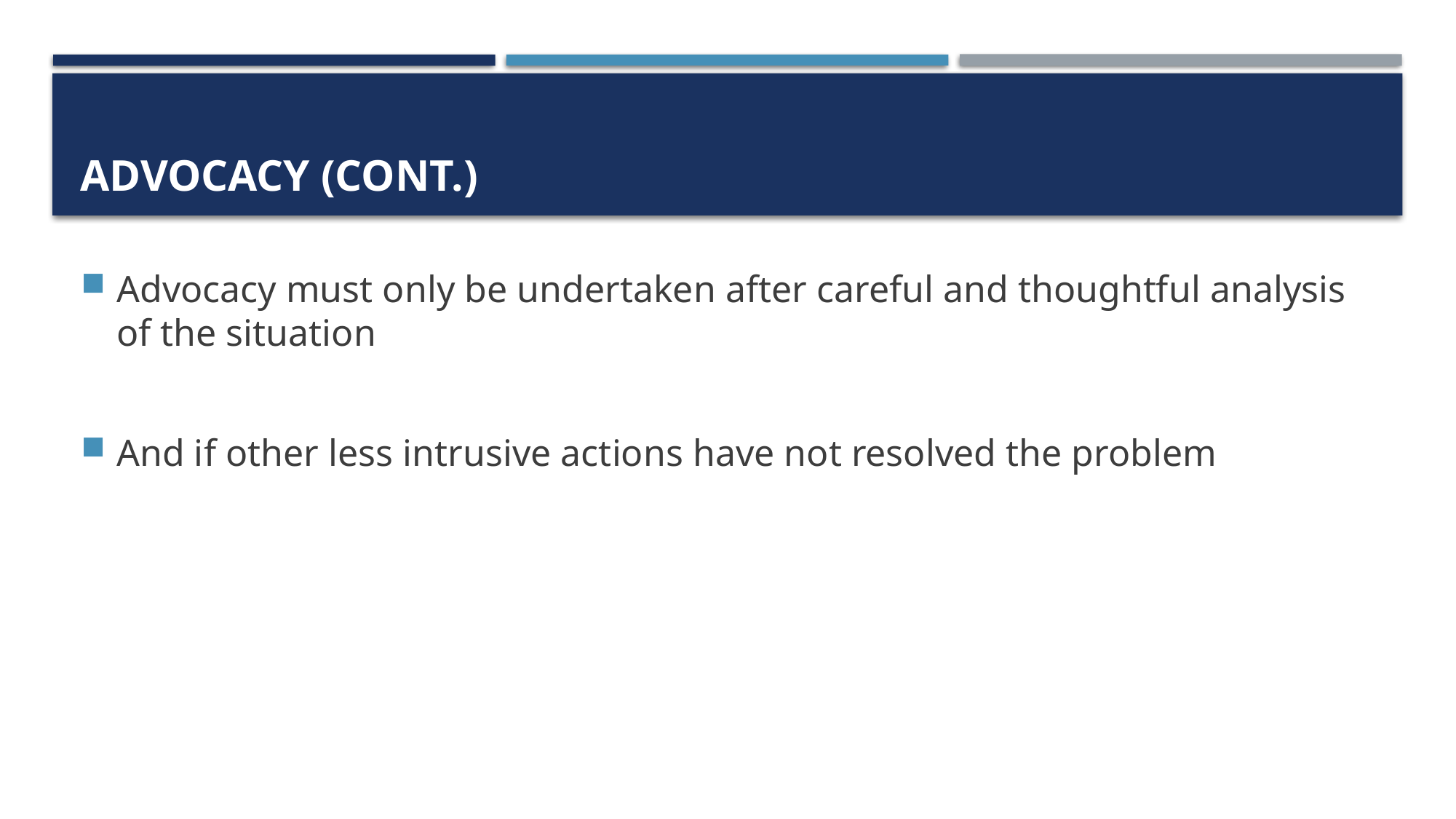

# Advocacy (cont.)
Advocacy must only be undertaken after careful and thoughtful analysis of the situation
And if other less intrusive actions have not resolved the problem

## Slide 28
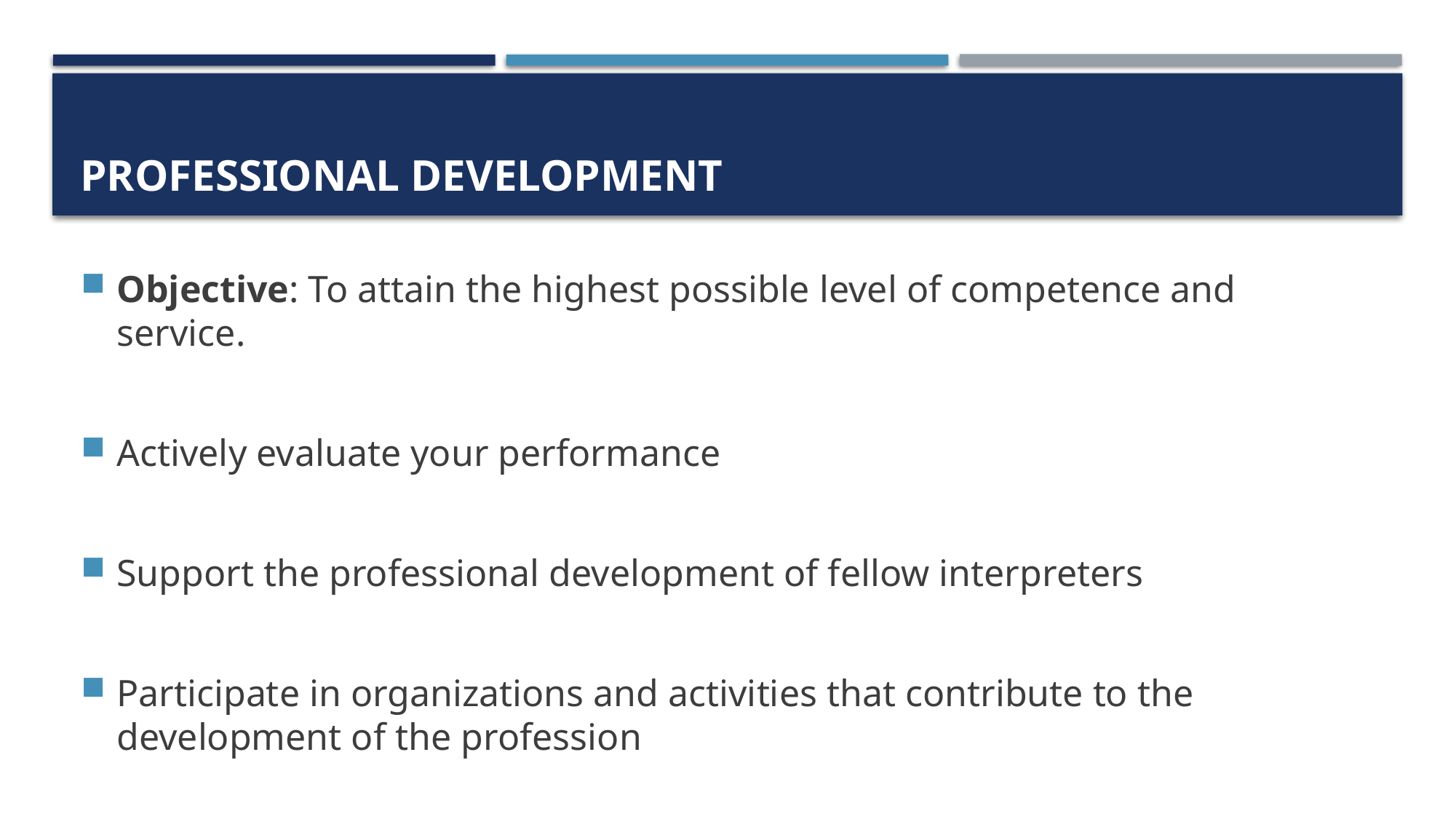

# Professional Development
Objective: To attain the highest possible level of competence and service.
Actively evaluate your performance
Support the professional development of fellow interpreters
Participate in organizations and activities that contribute to the development of the profession

## Slide 29
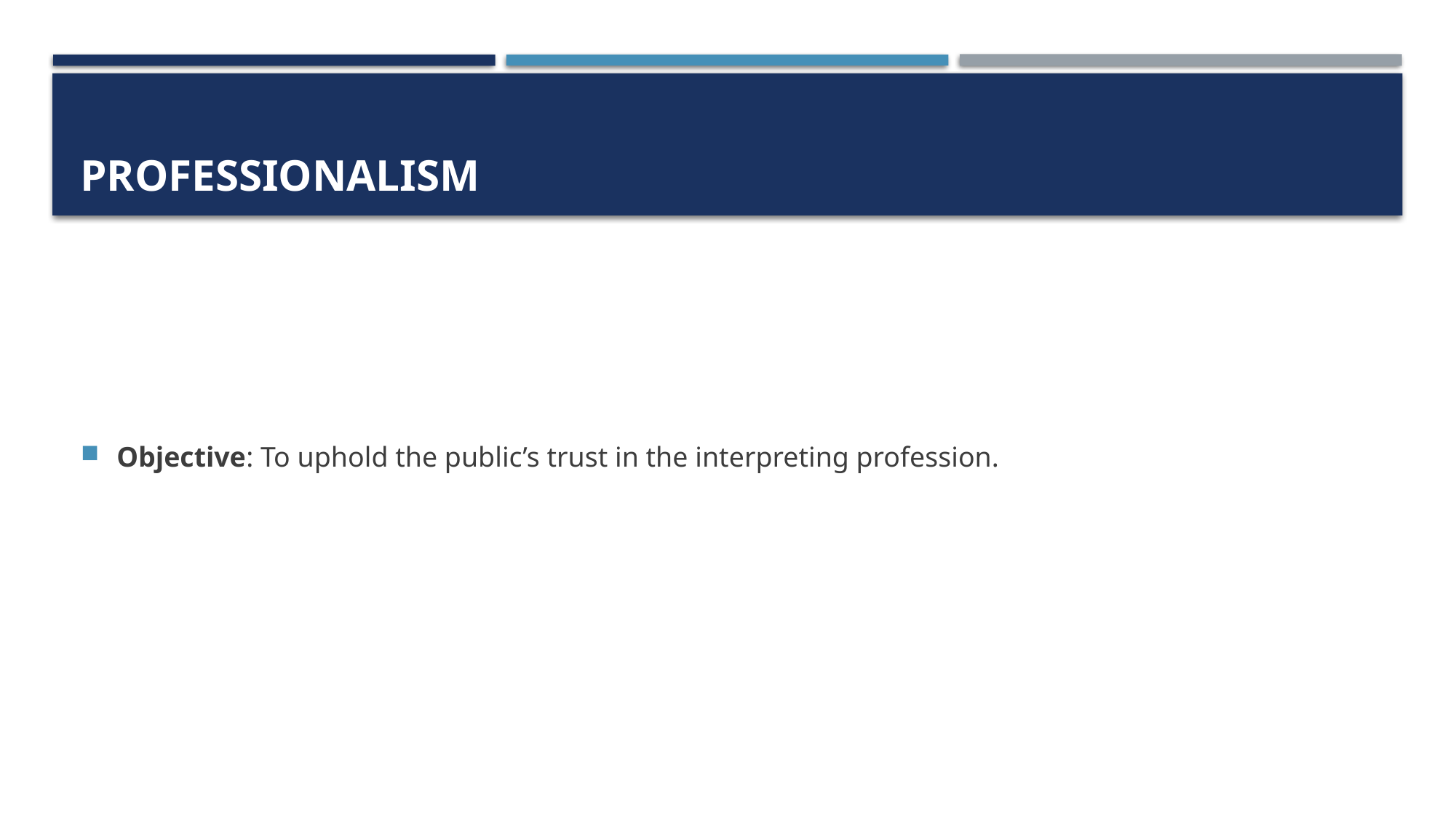

# Professionalism
Objective: To uphold the public’s trust in the interpreting profession.

## Slide 30
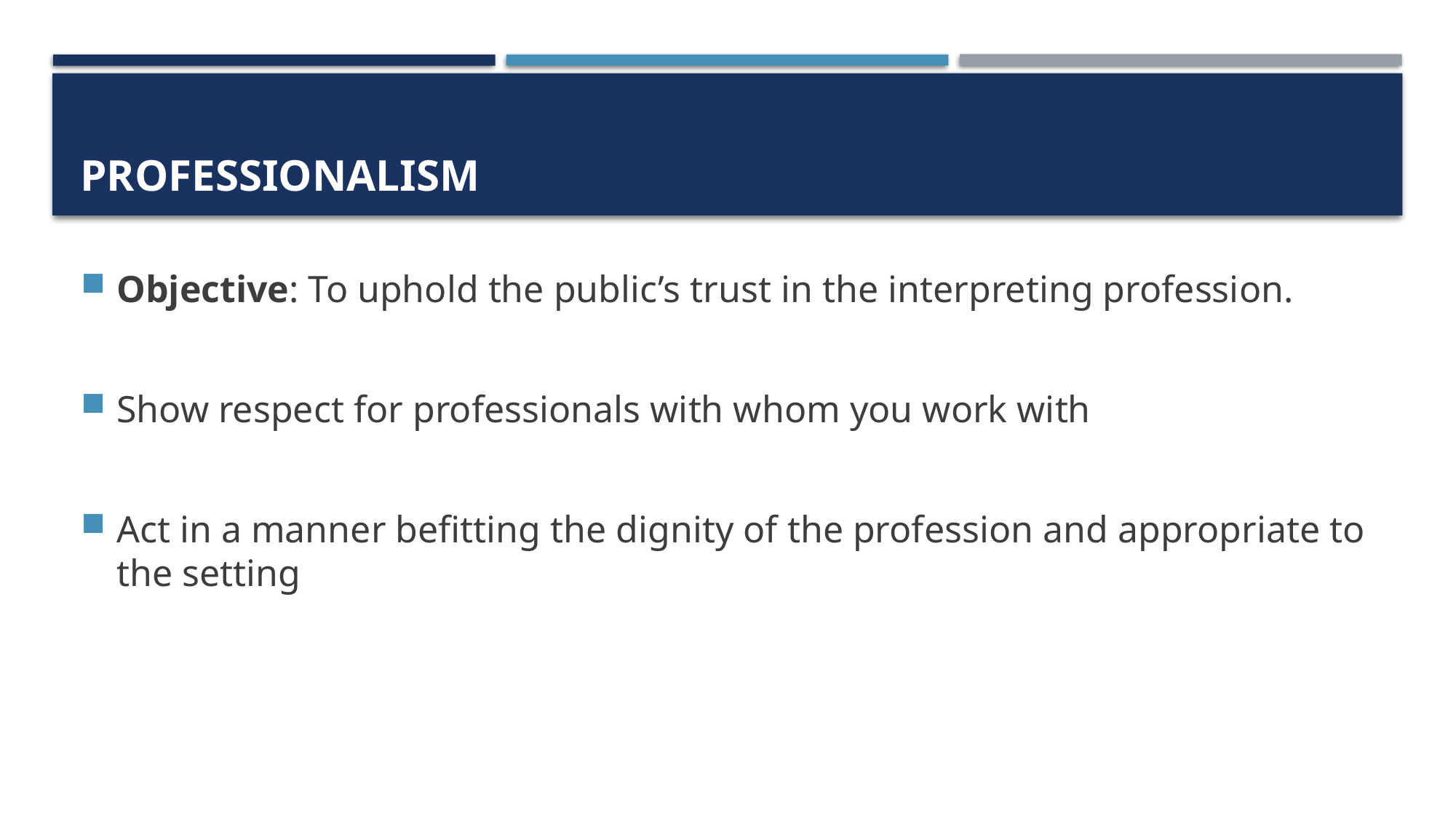

# Professionalism
Objective: To uphold the public’s trust in the interpreting profession.
Show respect for professionals with whom you work with
Act in a manner befitting the dignity of the profession and appropriate to the setting

## Slide 31
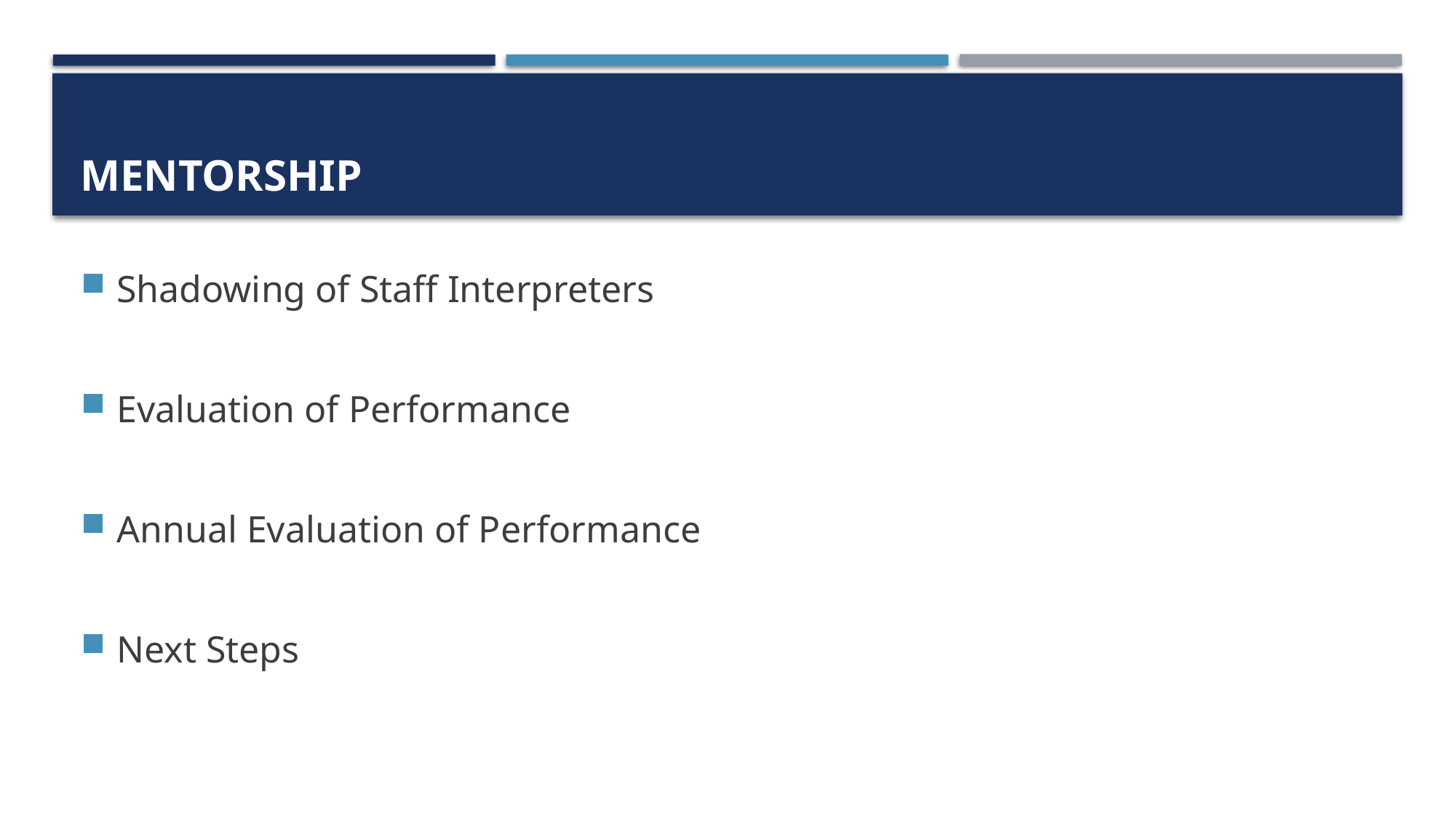

# Mentorship
Shadowing of Staff Interpreters
Evaluation of Performance
Annual Evaluation of Performance
Next Steps

## Slide 32
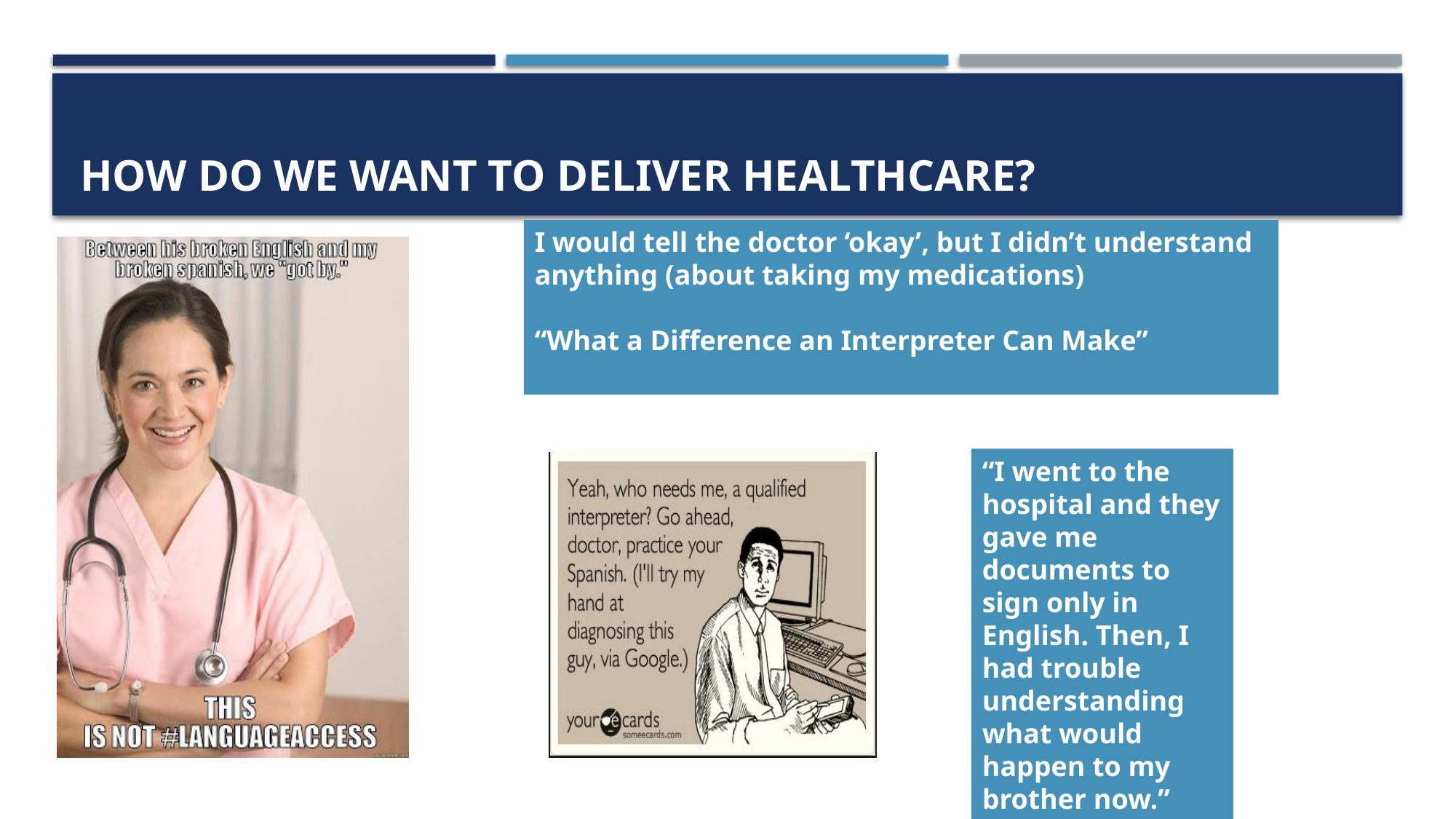

# How Do We Want To Deliver Healthcare?
I would tell the doctor ‘okay’, but I didn’t understand anything (about taking my medications)
“What a Difference an Interpreter Can Make”
“I went to the hospital and they gave me documents to sign only in English. Then, I had trouble understanding what would happen to my brother now.”
