# Supplementary material for: Overcoming the language barrier: a novel curriculum for training medical students as volunteer medical interpreters
Source: BMC Med Educ. 2022 Jan 10;22:27. doi: 10.1186/s12909-021-03081-0 (PMC8751325; doi:10.1186/s12909-021-03081-0)
Supplement: Supplementary file 3 — Additional file 3. Qualified Bilingual Staff Evaluation Form. [file 12909_2021_3081_MOESM3_ESM.pdf]

QBS Assessment Form

Colleagues Name: ________________________________ Language Pair: ___________
Evaluator Name: _________________________________ Date: __________________

The following items must be included in the introduction to Patient:

□ Name and current job title
□ Qualified to assist with simple interpretation
□ will attempt to interpret everything
□ will not add, omit or distort the message
□ may intervene to seek clarification or repetition
□ to ask questions
□ to speak directly to the provider
□ Name

The following items must be included in the introduction to provider:

□ Stated target language
□ Discussed accuracy and completeness
□ Discussed techniques on managing the flow
□ Directed the patient to speak directly to the provider
□ Introduction was complete
□ held a pre-session

Part II: Interpretation

1. Which mode of interpretation was mainly used? □ first-person mode □ third-person mode
   Note moments where the candidate lets the speaker go on for too long hence it affected their ability to interpret.
   ____
   ____

2. What techniques to manage the flow were applied?
   □ Hand gestures □ Immediate Interpreting □Verbal Queues

3. Fluency: Was the interpretation too fast, too slow, too soft, or too loud? Explain

Never	Occasionally	Usually	Always
□	□	□	□

   ____
   ____

4. When clarification was necessary, was the candidate transparent? If not, how was it handled?
   ____
   ____

5. Did the candidate clarify? Give an example of how they clarified
   ____
   ____

   If they did not clarify appropriately, how could they improve?
   ____

6. Did the candidate summarize?

Never	Occasionally	Usually	Always
□	□	□	□

7. Did the candidate switch between interpreting modes? If so, was it appropriate? Why or Why Not?

___

___

___

Part III: Completeness and Accuracy

Never	Occasionally	Usually	Always	
☐	☐	☐	☐	was the interpretation Accurate?
☐	☐	☐	☐	was everything interpreted (comments, counting)

List missed meaning, items omitted/ and or added:
If applicable, comment on any evaluator interventions.

___

___

___

___

Cultural Brokering:
Briefly describe any pertinent situations and what clarification steps were taken.

___

___

___

Part IV: Qualities of the Interpreter

Yes	No	
☐	☐	Did candidate take action to avoid personal conversation with the patient?
☐	☐	Was the candidate dress appropriately?
☐	☐	Was the candidate’s positioning appropriate? (If it should improve, please comment)

___

___

Part V: Comments

In what areas does the Interpreter need to improve?

☐ Introduction ☐ Accuracy ☐ Completeness ☐ Fluency ☐ Managing Flow
☐ Transparency ☐ Medical Terminology ☐ Self- Confidence ☐ Flexibility

Further Recommendations:

___

___

___
