# Supplementary material for: Overcoming the language barrier: a novel curriculum for training medical students as volunteer medical interpreters
Source: BMC Med Educ. 2022 Jan 10;22:27. doi: 10.1186/s12909-021-03081-0 (PMC8751325; doi:10.1186/s12909-021-03081-0)
Supplement: Supplementary file 4 — Additional file 4. Interpreter Training Post-Test. [file 12909_2021_3081_MOESM4_ESM.pdf]

Interpreter Training Exercise

Post-Test

Name: ________________________________

Date: _________________________

Post-Test Assessment

1. During the encounter, the attending doctor holds a side conversation with one of his residents. The patient looks at you, the interpreter, expecting for you to interpret the doctors' conversation, briefly explain how you should handle this situation?

2. Describe one of the principles of the interpreter code of ethics?

3. Why should the interpreter maintain the register of the speaker when interpreting?

4. List the barriers to interpreting in healthcare.

5. List the steps to intervening with transparency.

1. How comfortable do you feel in your interpreter skills?
    - ➢ Uncomfortable
    - ➢ Somewhat comfortable
    - ➢ Neutral
    - ➢ Somewhat comfortable
    - ➢ Very comfortable

2. How familiar are you with the Interpreter Code of Ethics?
    - ➢ Unfamiliar
    - ➢ Somewhat unfamiliar
    - ➢ Neutral
    - ➢ Familiar
    - ➢ Very familiar

3. How familiar are you with the concept of Intervening with Transparency?
    - ➢ Unfamiliar
    - ➢ Somewhat unfamiliar
    - ➢ Neutral
    - ➢ Familiar
    - ➢ Very familiar
