# Supplementary material for: Overcoming the language barrier: a novel curriculum for training medical students as volunteer medical interpreters
Source: BMC Med Educ. 2022 Jan 10;22:27. doi: 10.1186/s12909-021-03081-0 (PMC8751325; doi:10.1186/s12909-021-03081-0)
Supplement: Supplementary file 5 — Additional file 5. Example Qualified Bilingual Staff ID Badges. [file 12909_2021_3081_MOESM5_ESM.pdf]

AD·MAJOREM·DEI·GLORIAM 1870

LOYOLA MEDICINE

QUALIFIED BILINGUAL STAFF

Level 1

LOYOLA MEDICINE

QBS Level 1

CUSTOMER SERVICE

Ability to provide services in the target language where knowledge of medical terminology is NOT required.

EXAMPLE:

- Registration
- Appointment scheduling
- Pharmacy refill
- Blood draw/lab
- Dietary

Understand your limitations.

Request an interpreter when you don't feel comfortable interpreting specific vocabulary/terminology.

LOYOLA MEDICINE

QUALIFIED BILINGUAL STAFF

Level 2

LOYOLA MEDICINE

QBS Level 2

QUALIFIED BILINGUAL STAFF

Ability to provide services in the target language in various healthcare settings.

EXAMPLE:

- Routine return visit
- Triage
- Forms completion
- Radiology

REFRAIN FROM INTERPRETING DURING:

- Informed consents
- Delicate encounters
- New diagnosis encounters
- Palliative care
- Speech therapy
- Encounters that suddenly become complicated
- Encounters involving DCFS
- Encounters involving the police

Understand your limitations.

Request an interpreter when you don't feel comfortable interpreting specific vocabulary/terminology.
