# Supplementary material for: Overcoming the language barrier: a novel curriculum for training medical students as volunteer medical interpreters
Source: BMC Med Educ. 2022 Jan 10;22:27. doi: 10.1186/s12909-021-03081-0 (PMC8751325; doi:10.1186/s12909-021-03081-0)
Supplement: Supplementary file 6 — Additional file 6. Description of QBS Level 1 vs. Level 2. [file 12909_2021_3081_MOESM6_ESM.pdf]

QBS Level 1 – Customer Service	QBS Level 2 – Qualified Bilingual Staff
Description:<br><br>Ability to provide services in the target language where knowledge of medical terminology is not required.	Description:<br><br>Ability to provide services in the target language in various healthcare settings.
Examples:<br>• Registration/Admitting<br>• Appointment Scheduling<br>• Pharmacy refill<br>• Blood Draw/Lab<br>• Dietary	Example:<br>• Routine Return Visit<br>• Triage<br>• Forms Completion<br>• Radiology
Understand your limitations; request an interpreter when you don't feel comfortable interpreting specific vocabulary/terminology.	Refrain from interpreting during:<br>• Informed Consents<br>• Delicate encounters<br>• New Diagnosis encounters<br>• Palliative Care<br>• Speech Therapy<br>• Encounters that suddenly become complicated<br>• Encounters involving DCFS<br>• Encounters involving the Police<br><br>Understand your limitations; request an interpreter when you don't feel comfortable interpreting specific vocabulary/terminology.
