# Supplementary material for: Overcoming the language barrier: a novel curriculum for training medical students as volunteer medical interpreters
Source: BMC Med Educ. 2022 Jan 10;22:27. doi: 10.1186/s12909-021-03081-0 (PMC8751325; doi:10.1186/s12909-021-03081-0)
Supplement: Supplementary file 7 — Additional file 7. Interpreter Pre & Post Test Free Response Grading Rubric. [file 12909_2021_3081_MOESM7_ESM.pdf]

Interpreter Survey Free Response Rubric

Instructions

1. Please grade one survey at a time in accordance with the rubric.
2. Please grade one question at a time from each survey. For example, please grade all of the Question #1's from each survey before moving to Question #2.
3. If a participant's response does not match exactly with the rubric as we have designed it, please use your best judgment as a professional interpreter in assigning the response a grade.

Questions

1. During the encounter, the attending doctor holds a side conversation with one of his residents. The patient looks at you, the interpreter, expecting for you to interpret the doctors' conversation, briefly explain how you should handle this situation?
2. Describe one of the principles of the interpreter code of ethics?
3. Why should the interpreter maintain the register of the speaker when interpreting?
4. List the barriers to interpreting in healthcare.
5. List the steps to intervening with transparency.

CORRECT RESPONSES

Question	Correct Answer/Key Words
1	● Inform both parties that everything that is stated during the encounter will be interpreted.
2	Potential responses, each of which is included in the interpreter code of ethics:<br>● The interpreter treats as confidential, within the treating team, all information learned in the performance of their professional duties, while observing relevant requirements regarding disclosure.<br>● The interpreter strives to render the message accurately, conveying the content and spirit of the original message, taking into consideration its cultural content.<br>● The interpreter strives to maintain impartiality and refrains from counseling, advising or projecting personal biases or beliefs.<br>● The interpreter maintains the boundaries of the professional role, refraining from personal involvement.<br>● The interpreter continuously strives to develop awareness of his / her own and other (including biomedical) cultures encountered in the performance of their professional duties.<br>● The interpreter treats all parties with respect.<br>● When the patient's health, well-being, or dignity is at risk, the interpreter may be justified in acting as an advocate.<br>● The interpreter strives to continually further his / her knowledge and skills.<br>● The interpreter must at all times act in a professional and ethical manner.
3	Providers use the interpreter's representation of what has been said as a diagnostic tool. Given this, interpreters need to be mindful that any piece of information may be an important data source. To omit or distort any of the information could, therefore, result in serious clinical consequences.

**4**	**Linguistic Barriers**<br>• Word Choice<br>• Accents<br>• Medical Terminology<br><br>**Personal Barriers**<br>• Concentration<br>• Emotions<br>• Religion<br>• Exhaustion<br><br>**Setting Barriers**<br>• Hospital vs. Clinic<br>• Noise<br>• Patient Attitude<br>• Smells
**5**	1. Signal that you will stop interpreting.<br>a. Identify your voice as your own.<br>b. Say it in both languages.<br>c. Refer to yourself as “the interpreter” while intervening.<br>2. Explain to both parties the reasons why you are intervening so that everyone knows what is being said.<br>a. Being *transparent* means that if you have a valid reason to talk to one party, tell the other party what you will do.<br>b. Being *transparent* means everyone knows what is being said.<br>3. Once you’re done intervening quickly go back to the message converter role.

GRADING RUBRIC

**Question 1 (0 to 2)**		
**0 out of 2**	**1 out of 2**	**2 out of 2**
No response	Partially correct	Correct response

**Question 2 (0 to 1)**	
**0 out of 1**	**1 out of 1**
No response/incorrect response	The response is in the interpreter code of ethics

**Question 3 (0 to 3)**			
**0 out of 1**	**1 out of 3**	**2 out of 3**	**3 out of 3**
No response/<br>No argument made	Argument is weak and not in line with the correct response	Argument is vague, but in line with the correct response	Argument is clear and well-supported and in line with the correct response

Question 4 (0 to 3)			
**0 out of 3**	**1 out of 3**	**2 out of 3**	**3 out of 3**
No response/ No barriers to interpreting in healthcare listed	Lists at least one barrier to interpreting in healthcare which falls under one of the three categories of barriers.	Lists at least one barrier to healthcare which falls under two of the three categories of barriers.	Lists at least one barrier to healthcare which falls under all three of the categories of barriers.

Question 5 (0 to 3)			
**0 out of 3**	**1 out of 3**	**2 out of 3**	**3 out of 3**
No response/ No keywords present	1 step of intervening with transparency is correctly listed	2 steps of intervening with transparency are correctly listed	All 3 steps to intervening with transparency are correctly listed
